# Confronting Melanoma Radioresistance: Mechanisms and Therapeutic Strategies

**DOI:** 10.3390/cancers17162648

**Published:** 2025-08-14

**Authors:** Ielizaveta Gorodetska, Alexander Schulz, Gerhard Behre, Anna Dubrovska

**Affiliations:** 1 OncoRay—National Center for Radiation Research in Oncology, Faculty of Medicine and University Hospital Carl Gustav Carus, Technische Universität Dresden and Helmholtz-Zentrum Dresden-Rossendorf, 01307 Dresden, Germany; a.dubrovska@hzdr.de; 2 Institute of Radiooncology—OncoRay, Helmholtz-Zentrum Dresden-Rossendorf (HZDR) Dresden, 01328 Dresden, Germany; 3 Dessau Medical Center, Clinic for Internal Medicine I—Gastroenterology, Hematology, Oncology, Hemostaseology, Palliative Medicine, Infectious Diseases, Pneumology, Nephrology, 06847 Dessau-Rosslau, Germany; alexander.schulz@klinikum-dessau.de (A.S.); gerhard.behre@klinikum-dessau.de (G.B.); 4 German Cancer Consortium (DKTK), Partner Site Dresden, 01307 Dresden, Germany; 5 German Cancer Research Center (DKFZ), 69120 Heidelberg, Germany; 6 National Center for Tumor Diseases (NCT) Partner Site Dresden, 01307 Dresden, Germany

**Keywords:** melanoma, radiation therapy, resistance, metastasis

## Abstract

Melanoma is a highly aggressive skin cancer with survival rates varying significantly based on stage and genomic characteristics. While localized melanoma has favorable outcomes, metastatic melanoma is associated with poor prognosis and limited treatment options. Radiotherapy (RT), one of the most commonly used cancer treatments, is less effective in melanoma due to its intrinsic radioresistance. This review discusses the current knowledge about the biological mechanisms contributing to melanoma radioresistance, including the role of cancer stem cells (CSCs), DNA repair mechanisms, hypoxia, altered metabolism, and melanin production. It also examines preclinical and clinical studies on novel therapeutic approaches, such as targeting CSC pathways, inhibiting DNA repair, modulating hypoxia-induced metabolic shifts, and combining RT with immunotherapies or targeted therapies. Promising strategies, such as RT-induced immune responses and advanced RT techniques, show the potential to overcome resistance. However, melanoma’s heterogeneity and the limited clinical validation of these approaches remain significant challenges. Integrated therapeutic strategies targeting the multifaceted mechanisms of melanoma radioresistance are essential to improve treatment outcomes. Further clinical validation and personalized approaches are needed to address the heterogeneity of melanoma and enhance the efficacy of novel interventions.

## Simple Summary

Melanoma resistance to radiation therapy can be attributed to both intrinsic and acquired resistance mechanisms, such as enhanced DNA repair, hypoxia, and cancer stem cell activity, and is associated with treatment failure. Uncovering the molecular mechanisms of melanoma radioresistance may provide a foundation for more efficient and personalized melanoma therapies, reduce recurrence rates, and inspire further research into overcoming resistance in other radioresistant cancers, ultimately benefiting the broader oncology research community. This review primarily focuses on cutaneous melanoma, which represents the most common and extensively studied form of melanoma. Although the primary emphasis is on cutaneous melanoma, uveal melanoma is mentioned in rare instances as an example to illustrate specific concepts or phenomena, such as the unique characteristics of radiotherapy in this distinct melanoma subtype.

## 1. Introduction

Malignant melanoma is a type of skin cancer that starts in cells called melanocytes and spreads locally, regionally, and distantly [1]. It remains a significant global health issue, with increasing incidence rates. The global incidence is estimated at 2–3 cases per 100,000 people, with higher rates in certain regions like Australia and North America [2]. In Europe, melanoma incidence averages around 25 cases per 100,000, with Northern European countries reporting the highest rates. The survival rates have improved due to advancements in early detection and treatment, but discrepancies exist across different countries. In Germany, the incidence of melanoma has been rising, with rates around 18.2 cases per 100,000. The mortality rate is approximately 2.3 deaths per 100,000. The introduction of skin cancer screening has contributed to improved survival rates, although challenges remain in managing the disease effectively [3].

The 5-year survival rate for patients with localized melanoma can be as high as 85–95% [4]. Among different diagnostic parameters, Breslow thickness measures the depth of the melanoma tumor from the surface of the skin to the deepest point of the tumor cells and is used to determine the stage and prognosis of melanoma skin cancer [5]. For patients with thicker melanomas (Breslow thickness >3 mm), the survival rate decreases significantly, to approximately 50% for those with thicknesses between 1.51 and 3.00 mm [6,7].

Metastatic melanoma is characterized by its aggressive nature and ability to spread to various organs. Key characteristics include the following: (1) hematogenous spread, when melanoma cells disseminate through the bloodstream, leading to distant metastases [8]; (2) premetastatic niche formation, when tumor-derived extracellular vesicles and tumor secretory factors (e.g., chemokines and cytokines) prepare distant organs for metastasis by activating proinflammatory signaling [9,10]; and (3) variable clinical presentation with diverse symptoms depending on the organs involved [11]. Affected organs for distant metastases could include the lymph nodes [12], lungs [13], liver [14], brain [15], bone [16], skin [17], and gastrointestinal tract [18]. The prognosis for metastatic melanoma is much poorer, with a 5-year survival rate ranging from 5% to 19% depending on the location and number of metastases [19]. The median survival time for patients with metastatic melanoma is approximately 7.5 months, with some studies reporting a median overall survival (OS) of 5.3 months for those with distant metastases [20]. Overall, early detection and treatment significantly improve survival outcomes in malignant melanoma, while metastatic melanoma remains a challenging condition with limited treatment options.

Traditionally, melanoma subtypes have been categorized based on their site of origin. Malignant melanoma presents in the following four primary forms based on its origin: cutaneous melanoma (CM), from non-glabrous skin; acral melanoma (AM), from the palms, soles, and nail beds; mucosal melanoma (MM), the least common, from mucosal linings; and uveal melanoma (UM), from the eye’s uveal tract [21].

Melanomas can be further classified into four genomic subtypes based on the presence of specific driver mutations, namely B-Raf Proto-Oncogene (BRAF)-mutant, neuroblastoma RAS viral oncogene homolog (NRAS)-mutant, neurofibromatosis type I (NF1)-loss, and triple wild-type (TWT) [22]. Scientists sometimes distinguish the KIT Proto-Oncogene (KIT) melanoma subtype, which is primarily associated with acral and mucosal melanomas and is more prevalent in Asian populations. KIT mutations occur in approximately 10–20% of these melanoma subtypes, with a notable incidence of 10.8% in a study of 502 cases. These mutations are linked to shorter OS compared to patients without KIT alterations, indicating that KIT mutations serve as an adverse prognostic factor in melanoma [23,24]. Targeted therapies, like imatinib, have shown some efficacy in patients with KIT mutations, but responses can be limited and vary based on the specific mutation present [25,26]. Overall, KIT mutations are a critical factor in the prognosis and treatment strategies for specific melanoma subtypes.

Approximately 46–48% of metastatic melanoma cases harbor BRAF mutations, with a single point mutation at codon 600 (V600E) being the most common (70–88%), followed by V600K (10–20%) [27]. The median survival for BRAF-mutant patients not treated with inhibitors is 5.7 months, compared to 8.5 months for BRAF wild-type patients. However, those treated with BRAF inhibitors have significantly improved outcomes, with one-year survival rates reaching 83% [28,29]. In patients with brain metastases, BRAF mutation status is an independent prognostic factor, correlating with worse OS [30]. These findings highlight the importance of understanding BRAF mutation status in determining treatment strategies and prognostic outcomes in melanoma.

NRAS mutant melanoma accounts for approximately 15–20% of melanoma cases and is characterized by a more aggressive clinical course compared to BRAF mutant melanoma. NRAS mutant melanoma is associated with significantly worse progression-free survival (PFS) compared to NRAS wild-type melanoma, with hazard ratios indicating a higher risk of progression [31]. Although some studies show a trend towards shorter OS in NRAS mutant patients, results are mixed, and further research is needed to clarify these outcomes [32,33]. While some studies suggest NRAS mutations may confer a better response to immune checkpoint inhibitors (ICIs), others indicate no significant differences in overall response rates or survival compared to NRAS wild-type [33,34]. All in all, NRAS mutant melanoma presents unique challenges in treatment and prognosis, prompting a need for novel and more effective therapies.

The NF1-loss melanoma subtype is characterized by distinct clinical and biological features. Melanomas with NF1 mutations exhibit a higher mutational burden and a strong UV mutation signature. Patients with NF1-mutant melanoma tend to be older at diagnosis and predominantly male. This subtype is associated with significantly poorer survival outcomes, including a 1.9-fold increased risk of death from melanoma and a 2.0-fold increased risk of poor OS compared to other subtypes, even after adjusting for age and gender [35]. NF1 loss is linked to RAS activation and dependence on the MEK pathway, making these tumors potentially sensitive to MEK inhibitors, like trametinib. However, NF1 loss can also lead to resistance to RAF inhibitors, complicating treatment options [36]. Additionally, individuals with NF1 loss have a higher incidence of melanoma, with earlier diagnoses and thicker tumors, further emphasizing the need for cautious monitoring in this population [37].

Lastly, the TWT melanoma subtype is characterized by the absence of mutations in the three most common melanoma driver genes, namely BRAF, NRAS, and NF1. This subtype is associated with a lower mutational burden compared to other melanoma types, which can complicate treatment strategies, as TWT melanomas often do not respond well to targeted therapies that are effective in BRAF or NRAS mutant melanomas. Survival outcomes for TWT melanoma patients tend to be poorer compared to those with BRAF mutations. TWT patients exhibit a higher mortality risk, particularly among males and older individuals [38]. The prognosis for TWT melanoma can also be influenced by such factors as tumor location and stage at diagnosis, with acral melanoma showing particularly poor outcomes due to delayed diagnosis and the intrinsic characteristics of the tumor [39].

Melanoma can be treated using a combination of modalities, such as surgical resection, chemotherapy, immunotherapy, RT, and targeted therapy. Treatment strategies may involve single agents or combinations of therapies tailored to the individual patient’s health status, stage of disease, and tumor location [40]. The primary treatment for localized melanoma is wide local excision, which aims to remove the tumor along with a margin of healthy tissue. Surgery is often curative for early-stage melanomas [41]. While cytotoxic chemotherapy has been historically used to treat cutaneous melanoma, its current clinical utility is limited due to the advancement of more effective therapies, like ICIs and BRAF/MEK inhibitors. Chemotherapy offers lower objective response rates compared to other supportive care options, while often being associated with significant treatment-related toxicities that can adversely impact patient quality of life (QoL) [42]. As mentioned above, agents like ICIs (e.g., pembrolizumab, nivolumab) enhance the immune response against melanoma cells, particularly in advanced stages [43].

Targeted therapy for melanoma has significantly advanced treatment options, particularly for patients with specific genetic mutations. Medications like vemurafenib and dabrafenib target the BRAF (V600E) mutation and have shown improved OS in clinical trials, and they are often used as first-line treatments for metastatic BRAF-mutant melanoma [44]. MEK inhibitors (e.g., trametinib) are often used in combination with BRAF inhibitors, as they enhance treatment efficacy and help delay resistance mechanisms [45]. Numerous research efforts are currently focused on developing targeted therapies for melanomas driven by NRAS mutations, NF1 loss-of-function mutations, and other genetic alterations that activate the MAP kinase pathway [46]. Despite the effectiveness of targeted therapies, many patients develop resistance, requiring ongoing research into new treatment strategies and combination therapies [47]. Overall, targeted therapies represent a crucial component of personalized treatment for melanoma, particularly for genetically defined subtypes.

This review delves into the critical role of RT in the management of melanoma patients, emphasizing its evolving significance in treatment protocols. It provides a comprehensive exploration of RT applications for melanoma patients, highlighting its potential to target localized and metastatic disease. The review also examines the underlying mechanisms of radiation resistance, including intrinsic factors such as DNA repair efficiency, hypoxia, and tumor heterogeneity, as well as acquired resistance driven by adaptive cellular responses and microenvironmental influences [48]. Furthermore, it discusses mechanisms of radiation sensitivity, such as radiosensitization through targeted therapies and immune modulation. Finally, the review outlines innovative strategies to overcome resistance, including combination therapies with immune checkpoint inhibitors, DNA repair inhibitors, and metabolic modulators. By addressing these aspects, the review underscores the importance of integrating RT into multimodal treatment approaches for melanoma.

## 2. Radiotherapy

Radiotherapy (RT) is a treatment modality that uses high-energy radiation beams to eliminate cancer cells. RT offers a targeted approach, focusing radiation on the tumor site while minimizing damage to surrounding healthy tissues. Unlike local treatments, like surgery, which physically removes the tumor, RT targets cancer cells throughout the body or in close proximity to the tumor. There are two main types of RT, namely external and internal beam radiation. External beam RT (EBRT) delivers radiation from an external machine named a linear accelerator (LINAC) [49]. The most common form of RT is photon beam therapy, which typically employs high-energy X-rays or gamma rays to target cancer cells while sparing nearby healthy tissue [50]. Proton RT employs proton particles, which deposit most of their energy at a specific depth (known as the Bragg peak), minimizing damage to surrounding tissues [51]. FLASH RT is a specific technique within EBRT characterized by the delivery of ultra-high dose rates of radiation (>40 Gy/s) in milliseconds, reducing normal tissue toxicity while maintaining tumor control [52]. Microbeam radiotherapy (MRT) is an emerging treatment modality for melanoma, utilizing high-intensity, spatially fractionated X-ray beams. This technique delivers ultra-high doses in narrow microbeams, sparing surrounding healthy tissue while effectively targeting tumors. MRT has shown promise in overcoming melanoma radioresistance, attributed to its ability to induce localized tumor ablation and enhance anti-tumor immune responses [53]. Brachytherapy is a form of internal RT where radiation sources, such as seeds, ribbons, or capsules, are placed inside the body or near the tumor [54].

RT regimens can significantly influence therapeutic outcomes in melanoma, particularly when combined with systemic therapies. High-dose-per-fraction RT, such as stereotactic body radiotherapy (SBRT) and hypofractionation, has been shown to enhance local tumor control and systemic immune responses. For example, SBRT can induce immunogenic cell death, releasing tumor antigens and promoting T-cell activation, which synergizes with immune checkpoint inhibitors (ICIs) to elicit systemic anti-tumor immunity, including abscopal effects [55]. Additionally, RT can modulate the tumor microenvironment by reducing hypoxia and enhancing immune infiltration, further improving the efficacy of combination therapies [56]. These findings underscore the potential of optimized RT regimens to overcome radioresistance and improve outcomes in melanoma.

RT is primarily utilized in specific, well-defined clinical scenarios, including cases of medical inoperability and certain melanoma subtypes, such as lentiginous melanoma [57], mucosal melanoma [58], uveal melanoma [59,60,61], and ocular melanoma [62]. Adjuvant RT following lymphadenectomy in node-positive melanoma patients has been shown to reduce local and regional recurrence rates [63,64]. However, its use remains controversial and underutilized, primarily due to the absence of demonstrated improvements in OS. Palliative radiotherapy (PRT) improves patient QoL by alleviating cancer-associated symptoms, such as painful bone metastasis, spinal cord compression, brain metastasis, or soft tissue metastasis causing pain, bleeding, or obstruction [65].

RT exerts its curative effects primarily through the generation of reactive oxygen species (ROS) and direct DNA damage. Ionizing radiation induces ROS via the radiolysis of water, leading to oxidative stress that damages cellular components, particularly DNA. This results in single- and double-strand DNA breaks (DSB), which overwhelm the repair mechanisms. The RT-induced damage triggers different types of cell fates, including apoptosis, mitotic catastrophe, ferroptosis, necrosis, autophagy, or senescence, leading to cell death or inhibition of cell proliferation [66,67].

While RT has shown some benefit in managing melanoma, its effectiveness is often limited by the inherent resistance of melanoma cells to radiation [68]. Cell culture studies initially suggested that melanoma exhibits high intrinsic radioresistance, characterized by a “shoulder” effect in cell survival curves [69,70,71,72]. This implies a high capacity for DNA repair and a potential advantage to delivering higher radiation doses per fraction. The clinical trials utilizing this approach with larger radiation doses supported these laboratory findings [73,74,75]. Enhancing the sensitivity of melanoma cells to RT could significantly expand its clinical applicability. Several factors contribute to melanoma radioresistance, including the cancer stem cell populations, tumor microenvironment (TME), activation of the DNA damage repair mechanisms, hypoxia, cellular metabolism, pigmentation, and melanin production.

## 3. Cancer Stem Cells

Cancer stem cells (CSCs) are a subpopulation of cells within tumors that possess unique properties, including self-renewal, differentiation, and resistance to therapies. In melanoma, CSCs contribute to RT resistance and promote tumor recurrence after treatment [76,77,78]. CSCs in melanoma are heterogeneous [79,80,81,82,83,84] and dynamically change as an evolutionary adaptation to environmental pressures, including hypoxia, immune surveillance, and therapy. These pressures drive clonal selection and phenotypic plasticity, enabling melanoma cells to survive, proliferate, and metastasize under stress [85,86,87]. Hypoxia in the tumor microenvironment activates hypoxia-inducible factors (HIFs), promoting stem-like properties such as self-renewal, metabolic reprogramming, and resistance to apoptosis. Melanoma cells with these traits are selected for survival in oxygen-deprived regions and enriched for CSC populations [88]. Immune surveillance exerts pressure on melanoma cells to evade detection. CSCs achieve this by downregulating antigen presentation, expressing immune checkpoint molecules (e.g., PD-L1), and secreting immunosuppressive factors, allowing them to escape immune attack and maintain their population [89]. Moreover, CSCs exhibit phenotypic plasticity, allowing non-CSCs to transition into CSCs under stress [90]. This adaptability ensures the maintenance of CSC populations and tumor heterogeneity, enabling melanoma to dynamically respond to environmental changes [91].

Melanoma CSCs can be identified by surface markers, such as cluster of differentiation (CD) 133, CD20, CD271, aldehyde dehydrogenases (ALDHs), and ATP-binding cassette sub-family B member 5 (ABCB5), which are associated with their tumorigenic potential and resistance to treatment. However, the specificity of these markers for CSC populations remains questionable [83,84,92,93]. A study by Quintana and co-workers demonstrated that none of the 22 putative melanoma CSC markers enriched tumorigenic cells [84]. A recent study by Speigl and co-authors analyzed putative CSC markers (ALDH1A1, ABCG2, CD44v7/8, CD44v10, CD133, CD271, and Nestin) in melanoma and revealed widespread expression across cell lines, early-passage strains, and patient tissues, suggesting limited specificity for CSCs. These proteins were also detected in non-malignant cells, such as melanocytes and fibroblasts, limiting their potential as CSC-specific bi-omarkers and therapeutic targets. Similar to the CSCs in many other tumor entities, melanoma CSC populations can be enriched in vitro by propagation and passaging under sphere-forming conditions [94]. It was also found that melanoma cancer cells with elevated autofluorescence exhibited characteristics of CSCs [95]. This autofluorescence resulted from riboflavin (vitamin B2) accumulation in the ATP-dependent transporter ATP-binding cassette superfamily G member 2 (ABCG2)-positive vesicles and can serve as a tool for isolating CSCs from tumor tissue [96]. Marzagalli et al. demonstrated that vitamin E derivative δ-tocotrienol (δ-TT) inhibited the autofluorescent population in A375 melanoma cells. The authors showed that melanoma cell lines A375 and BLM share the expression of the stem cell markers ABCB5 and CD44, but A375 cells uniquely expressed CD271 and exhibited melanosphere formation, a hallmark of stemness, unlike BLM cells [82,96]. This study confirmed the limitations of using single surface markers as specific indicators of CSC function, as their expression is shown to be variable depending on the analyzed models and experimental context.

The concepts of plasticity, heterogeneity, and transdifferentiation are particularly relevant when discussing melanoma CSCs, and they contribute significantly to the challenges in treating this aggressive cancer [83]. Melanoma is highly heterogeneous, with subpopulations of cells expressing distinct markers like CD271 and CD133, whose role as CSC identifiers remains questionable [97,98]. This heterogeneity arises from the neural crest (NC) origin of melanoma cells. The embryonic origin of melanoma cells and, in particular, the NC-associated transcriptional factors, provide melanoma with differentiation plasticity [99]. This is further influenced by the TME, genetic mutations, and epigenetic changes. Functionally, this diversity allows melanoma stem cells to adapt to environmental stresses, evade immune responses, and resist therapies, contributing to tumor progression and relapse [77]. CD271^+^ cells, for example, are associated with higher tumorigenicity and metastatic potential [81,100]. Melanoma stem cells exhibit phenotypic plasticity, allowing them to switch between proliferative and invasive states in response to environmental cues like hypoxia or nutrient deprivation. This plasticity contributes to drug resistance and metastasis, as cells adapt to therapy by entering slow-cycling, drug-resistant states. It is regulated by multiple signaling pathways, like WNT, AXL, microphthalmia-associated transcription factor (MITF), and nuclear factor kappa-light-chain-enhancer of activated B cells (NF-κB), allowing cells to adapt to changing conditions, evade therapies, and promote metastasis [98,101,102]. Melanoma stem cells exhibit transdifferentiation, which is defined as their ability to differentiate into cell lineages other than their original melanocytic lineage. This phenomenon is linked to their NC origin and inherent plasticity. Melanoma stem cells can transdifferentiate into vascular and neural lineages, contributing to processes including vasculogenic mimicry and tumor adaptability. Transdifferentiation is also observed in melanoma cells reprogrammed to pluripotent states [76,97]. Melanoma cells can also adopt endothelial-like properties, form vascular structures, or revert to a more aggressive phenotype under specific microenvironmental cues. This transdifferentiation plays a role in metastasis, therapy resistance, and tumor progression [103,104]. Melanoma vasculogenic mimicry (VM) can be understood as an ecological adaptation within a multi-dimensional spatiotemporal pathological ecosystem shaped by the interplay of external environmental pressures and internal competitive dynamics [105]. External pressures, such as hypoxia and nutrient deprivation, create a hostile microenvironment that forces melanoma cells to adopt endothelial-like properties, enabling the formation of vascular-like networks independent of traditional angiogenesis. These structures ensure tumor perfusion and survival under nutrient deprivation and hypoxic stress. Concurrently, internal dynamics, including cellular heterogeneity and metabolic demands, drive competition among tumor cells, favoring those capable of VM as a survival strategy [106,107].

Melanoma CSCs often harbor mutations in such genes as BRAF and NRAS, which are associated with resistance mechanisms, including the reactivation of mitogen-activated protein kinases (MAPK) signaling pathways [108]. They frequently express drug efflux pumps, which help them to expel therapeutic agents, including those used in RT, further enhancing their survival [109]. Melanoma CSCs also exhibit a slow-cycling phenotype, allowing these cells to evade the cytotoxic effects of conventional chemotherapy and RT, which primarily target rapidly dividing cells [110].

Melanoma CSC resistance is often linked to specific molecular pathways, such as the Hippo signaling pathway, which can sustain melanoma CSC survival and proliferation. Fisher et al. found elevated levels of yes-associated protein 1 (YAP1), tafazzin (TAZ), and TEA domain family member (TEAD) in BRAF inhibitor-resistant melanoma CSCs. This upregulation enhances cell survival, promotes spheroid formation, and increases Matrigel invasion and tumor formation [111]. Knowles and co-authors identified a rare CD24^+^CD271^+^ subpopulation within melanoma with heightened stem-like properties, including lineage plasticity and self-renewal. This hybrid population exhibited enhanced sphere formation, migration, and drug resistance compared to CD24^+^ or CD271^+^ subpopulations alone. The authors hypothesized that certain microenvironmental conditions, such as anti-cancer therapy or oxidative stress, can increase the functional demands for this CD24^+^CD271^+^ subpopulation, and therefore, melanoma CSCs can undergo phenotype switching, allowing them to adapt to therapeutic pressures and maintain tumor growth despite treatment [112].

CD44 and CD133 identify CSCs in melanoma but show varying expression levels and clinical significance. For instance, CD44 and ALDH1A1 expressions are significantly higher in melanoma than in other skin cancers, suggesting a unique role in melanoma aggressiveness [113]. However, the role of CD44 and CD133 as melanoma CSC markers remains controversial, with studies indicating that both CD133^+^ and CD133^−^ subsets can initiate tumors [80,114]. CD44, a transmembrane glycoprotein, is involved in cell adhesion, migration, and interaction with the extracellular matrix (ECM) through its binding to hyaluronic acid (HA) and inducing Id1/Id3 expression [115,116]. In melanoma, CD44 contributes to tumor invasion, metastasis, and resistance to therapy by promoting epithelial-to-mesenchymal transition (EMT) and enhancing cell survival under stress conditions. Additionally, CD44 signaling influences the tumor microenvironment by modulating immune evasion and angiogenesis. CD133, another transmembrane glycoprotein, is associated with stemness, self-renewal, and differentiation potential in melanoma cells. It is often linked with enhanced tumorigenicity, metastatic capacity, and resistance to chemotherapy. CD133^+^ melanoma cells exhibit increased survival under stress conditions, such as hypoxia, and are more likely to evade apoptosis, contributing to tumor progression and therapy resistance. Functionally, CD133 is thought to interact with signaling pathways, like WNT, Hedgehog, and Notch, which regulate stemness and plasticity [100,117]. It also activates the PI3K/AKT pathway, reduces apoptosis, and induces chemoresistance [118,119].

ALDH isozymes play a significant role in CSCs and their response to RT. ALDH^+^ melanoma cells have been identified as more tumorigenic than their ALDH^−^ counterparts. These cells exhibit characteristics of CSCs, including self-renewal and differentiation capabilities. Silencing ALDH1A, a key isozyme, has been shown to induce apoptosis and reduce tumorigenesis, suggesting that targeting ALDH could enhance treatment efficacy [120]. Dinavahi et al. have developed a novel multi-isoform ALDH inhibitor, KS100, which showed effectiveness in reducing melanoma tumor growth in preclinical models. This inhibitor was designed to target multiple ALDH isoforms, potentially overcoming the limitations of isoform-specific inhibitors [121]. Another approach involved using dendritic cell-based vaccines targeting ALDH^+^ CSC-like cells, which showed promise in reducing tumor recurrence and metastasis in melanoma models. The authors demonstrated that vaccination with ALDH^high^ SCC7 cancer stem cell-loaded dendritic cells (CSC-DCs) significantly reduced local tumor recurrence and prolonged survival in mice with established SCC7 tumors. This therapeutic effect was further enhanced by the concomitant administration of anti-PD-L1, an ICI. Moreover, in the D5 melanoma model, CSC-DC vaccination effectively inhibited primary tumor growth, reduced spontaneous lung metastases, and improved survival in mice [122].

Another putative CSC marker, the transmembrane transporter ABCB5, plays a significant role in radiation resistance and chemoresistance in melanoma. ABCB5 mediates melanoma therapy resistance through the efflux of chemotherapeutic drugs [123,124,125]. ABCB5 is implicated in the survival of melanoma-initiating cells, which are often resistant to therapies, including RT. Its expression is linked to a proinflammatory cytokine signaling circuit that helps maintain slow-cycling, chemoresistant cells, thereby promoting tumor growth and resistance to treatment [126]. ABCB5 induces metastasis through NFkB pathway activation by inhibiting p65 ubiquitination to enhance p65 protein stability [127]. Moreover, treatment, like temozolomide, can enrich ABCB5-expressing cells, suggesting that conventional therapies may inadvertently select for more resistant tumor cell populations [128]. Patients with ABCB5-positive tumors exhibited poorer OS rates compared to those with a negative expression. This suggests that ABCB5 could serve as a valuable prognostic factor in melanoma, helping to identify patients at higher risk for aggressive disease [129]. In BRAF inhibitor-resistant melanoma cell lines, ABCB5 is overexpressed, indicating its role in resistance mechanisms, although it may not be a primary targetable contributor [130]. Circulating tumor cells (CTCs) enriched for ABCB5 exhibit distinct transcriptomic profiles, suggesting that ABCB5^+^ CTCs may have an invasive phenotype and play different roles in disease progression compared to other melanoma subpopulations [131].

Targeting CSC-specific pathways and markers may help overcome resistance and improve treatment outcomes [132]. Understanding the role of CSCs in melanoma biology is crucial for developing effective therapies against this malignancy. The PI3K/Akt/mTOR signaling pathway is involved in cell survival and proliferation, plays a role in drug resistance in CSCs, and is a potential target for cancer therapies. Research is focusing on the role of CD133-dependent activation of the PI3K/mTOR pathway in melanoma stem-like cells, which contributes to drug resistance and tumor recurrence. Combining PI3K-AKT-mTOR inhibitors with other pathway inhibitors, such as MAPK/MEK/ERK inhibitors, is being explored to address the compensatory pathway activation that often leads to resistance [133]. Preclinical studies suggest that targeting ALDH1 could be an effective strategy for treating metastatic melanoma. ALDH1 inhibitors, such as diethylaminobenzaldehyde, combined with chemotherapy (e.g., dacarbazine), significantly reduce tumor growth and the number of residual tumorigenic cells in melanoma xenografts [134]. There have been some promising clinical trials targeting CSCs in melanoma. CD20 in melanoma was first reported to be enriched in CSCs [76]. One study involved the use of rituximab, an anti-CD20 therapeutic antibody, in combination with dacarbazine, to target CD20^+^ melanoma cells. This approach led to lasting remission in a chemotherapy-refractory metastatic melanoma patient, providing clinical evidence that targeting CSCs can produce regression in melanoma [135]. Additionally, cytokine-induced killer (CIK) cells have demonstrated effective activity against autologous metastatic melanoma, including cells with stemness features. This immunotherapy approach showed intense tumor-killing activity, moving clinical investigation closer to a new treatment for metastatic melanoma [136]. Ganglioside GD2 has emerged as a potential marker of melanoma CSCs and a promising therapeutic target. GD2 is a glycolipid expressed on the surface of melanoma cells, particularly those with stem-like properties, and is associated with tumorigenicity, metastasis, and therapy resistance. Its expression correlates with enhanced invasive potential and poor prognosis in melanoma. Bispecific antibodies (bsAbs) targeting GD2 and immune effectors (e.g., T cells) have shown preclinical efficacy by redirecting immune cells to GD2^+^ melanoma cells, enhancing tumor cell killing. This approach leverages the immune system to selectively target CSCs and reduce tumor burden [137,138]. GD2-specific chimeric antigen receptor T-cell therapy (CAR-T) cells have demonstrated potent anti-tumor activity in preclinical melanoma models. These engineered T cells selectively recognize and kill GD2^+^ melanoma cells, including CSCs, while sparing normal tissues with low GD2 expression. GD2-CAR therapy also shows promise in overcoming immune evasion and reducing metastasis [139].

Another study explored the use of Lunasin, a bioactive peptide, which showed significant therapeutic activity against melanoma by specifically targeting melanoma CSCs. Lunasin treatment reduced tumor growth in mouse xenografts and induced differentiation in melanoma CSCs, suggesting its potential as a novel therapeutic option for advanced melanoma [140]. Metabolic modulator phenformin has been investigated for its ability to target the CSC compartment in melanoma. It significantly reduced cell viability and growth in both CSC and non-CSC populations, suggesting its potential as an anti-cancer therapy [141]. MicroRNAs (miRNAs) have also been explored as a target for melanoma, with the results showing that miR-S8 could suppress the stemness of melanoma stem-like cells by targeting specific transcription factors, like Y-box binding protein 1 (YB-1). This approach has shown promise in preclinical models [142]. The development of CAR-based therapy for targeting melanoma CSC markers, such as CD133, ALDH1, and GD2, especially the strategy for dual- and multitargeting of different tumor antigens by CARs [143], holds significant promise for treating aggressive and therapy-resistant cancers. Strategies to improve the efficacy of CAR-T therapy for melanoma include enhancing T-cell trafficking to tumors by modifying chemokine receptors or using combination therapies to improve tumor infiltration. Overcoming the immunosuppressive TME is another critical focus, achieved through the co-administration of ICIs or engineering CAR-T cells to resist suppressive cues, like TGF-β, hypoxia, and tumor acidification [144]. Incorporating safety switches, such as suicide genes, helps mitigate toxicity and improve safety regarding potential off-target effects. Additionally, developing dual-targeting CARs that recognize multiple melanoma-specific markers can enhance specificity and reduce the risk of tumor escape. These approaches collectively aim to optimize CAR-T therapy for melanoma [145].

## 4. DNA Damage Repair Mechanisms

DSBs represent the most lethal form of damage induced by ionizing radiation. They initiate a cascade of cellular DNA damage responses (DDRs), including the activation of DNA damage detection and signaling pathways, induction of cell cycle arrest, and engagement of DNA repair mechanisms [146]. A key characteristic of the cellular response to DNA DSBs is the rapid accumulation and localization of various DNA repair and signaling proteins near the damaged site. This process begins with the phosphorylation of histone H2AX by ataxia telangiectasia-mutated (ATM) protein, leading to the formation of distinct nuclear puncta known as radiation-induced foci (RIF) [147]. DNA repair inhibitors have demonstrated efficacy in radiosensitizing melanoma, enhancing the therapeutic effects of RT by impairing the ability of tumor cells to repair radiation-induced DNA damage. The radiosensitization potential of the DNA-dependent protein kinase catalytic subunit (DNA-PKcs) inhibitor peposertib was assessed in patient-derived xenograft models of melanoma brain metastases. The authors proposed peposertib as a radiosensitizer for brain metastases and demonstrated a method for combining laboratory and drug behavior data to create effective RT plans [148]. CC-115, a dual inhibitor of mTOR and DNA-PK, enhanced radiosensitivity in melanoma by blocking DSB repair. This combination therapy significantly reduced tumor cell survival and clonogenicity in melanoma models [149]. Targeting poly ADP-ribose polymerase 1 (PARP-1), a key DNA repair enzyme, with inhibitors like olaparib sensitizes melanoma cells to RT. Combining PARP inhibition with RT results in synergistic effects, reducing tumor growth and delaying regrowth [150]. Another study showed that MAPK inhibition led to a synthetic lethal interaction when combined with PARP inhibitors, resulting in a significant reduction in melanoma cell growth in vitro and in vivo [151]. Giunta and colleagues have demonstrated that BRAFV600 mutant cell lines, even those with pre-existing or developed resistance to BRAF and MEK inhibitors, can be effectively targeted by the combined use of the ataxia telangiectasia and Rad3-related protein (ATR) inhibitor AZD-6738 and the PARP inhibitor olaparib [152]. Checkpoint Kinase 1 (CHK1) mediates cell cycle arrest to allow DNA repair. CHK1 inhibitors, such as prexasertib, abrogate this checkpoint, forcing melanoma cells to progress through the cell cycle with unrepaired DNA. This leads to mitotic catastrophe and radiosensitization [153]. Histone deacetylase (HDAC) inhibitors such as sodium butyrate radiosensitize melanoma cells by suppressing DNA repair activity. They reduce the expression of DNA repair proteins, like Ku70 and DNA-PK, leading to persistent DNA damage and increased radiation sensitivity [154]. The combination of ceralasertib (an ATR inhibitor) and durvalumab (an anti-programmed death ligand 1 (PD-L1) ICI) demonstrated a tolerable safety profile in patients with metastatic melanoma. This combination showed potential as a salvage therapy for immunotherapy-resistant melanoma, offering a new option for patients who have failed frontline immunotherapy [155]. A gene-profile signature associated with distant metastasis and poor prognosis in malignant melanoma reveals overexpression of DNA repair genes, particularly those involved in recovering stalled replication forks, in primary tumors with poor outcomes. This suggests that genetic stability is crucial for melanoma metastatic progression, potentially explaining its resistance to therapies and offering new drug discovery opportunities [156].

The protein p53 plays a role in the DNA repair process by halting the cell cycle. A transcription factor p53, often referred to as the “guardian of the genome”, governs the expression of genes critical to essential cellular functions, such as DNA repair, apoptosis, cell cycle regulation, and differentiation [157]. p53 is mutated in the majority of human cancers; however, in wild-type (WT) melanoma, p53 is found inactivated in approximately 90% of cases, with around 10–20% carrying disabling point mutations [158,159,160]. Melanoma cells often express small molecular weight variants of p53, such as p53*β* or Δ40p53, which can alter the function of WT p53. These variants may contribute to the resistance of melanoma to DNA-damaging chemotherapy by modulating p53-dependent transcription and apoptosis pathways [161]. p53 variants can significantly affect the response of melanoma cells to RT by altering the normal function of the p53 protein, which is crucial for DNA damage response and apoptosis. In melanoma, even when p53 is intact, its activation in response to DNA damage can be impaired, contributing to radioresistance. This impairment may be due to defects in the pathways that activate p53, such as the phosphorylation state of Ser-376, which is not regulated by DNA damage in some melanoma cell lines [162]. Furthermore, the presence of p53 mutations or variants can lead to an abnormal response to radiation, as these variants may not effectively induce apoptosis or cell cycle arrest, which are critical for the therapeutic effects of RT [163]. Understanding these mechanisms is essential for developing strategies to overcome radioresistance in melanoma.

The Fanconi anemia (FA) pathway coordinates a complex process involving elements of three key DNA repair pathways, namely homologous recombination (HR), nucleotide excision repair (NER), and mutagenic translesion synthesis (TLS). This mechanism is usually activated in response to genotoxic stress, including ionizing radiation. Kao and co-authors have demonstrated that FA DNA repair genes are transcriptionally upregulated in malignant melanoma compared to non-melanoma skin cancer (NMSC) [164]. This means that the FA pathway could contribute to melanomagenesis and resistance to chemotherapy and probably RT [165]. Consequently, FA genes represent promising targets for the development of innovative melanoma therapies.

The resistance of melanoma cells to DNA-damaging agents is influenced by several molecular mechanisms. One significant factor is the enhanced activity of HDACs, which can lead to resistance against alkylating drugs, like temozolomide. Inhibition of class I HDACs has been shown to sensitize melanoma cells to these agents by suppressing DNA DSB repair through HR, involving proteins like RAD51 and FANCD2 [166]. Moreover, melanoma cells can exhibit enhanced photoproduct repair and post-replication recovery, contributing to their resistance phenotype. This enhanced repair capability allows melanoma cells to improve the management of DNA damage, thereby increasing their resistance to chemotherapeutic agents [167]. The overexpression of mouse double minute homolog (MDM) 2 and MDM4 has been identified as a factor that inhibits p53 function in melanoma. Targeting these interactions to reactivate p53 is considered a viable therapeutic strategy, especially in combination with other treatments, like kinase inhibitors [168]. Amplifications of MDM2 and MDM4, which are negative regulators of p53, are associated with metastatic melanoma. Patients with these amplifications may benefit from MDM2 and MDM4 inhibitors, especially if they have wild-type TP53 [169]. Furthermore, small molecules, like MJ25, have been identified as potential p53 activators, showing cytotoxic effects on melanoma cells by inhibiting thioredoxin reductase 1 (TrxR1) and enhancing p53-dependent transactivation [170]. High expression levels of p21, a p53 target gene, are associated with increased sensitivity to targeted therapies, such as BRAF inhibitors and MDM2 inhibitors. This suggests that p21 expression could serve as a predictive biomarker for the efficacy of p53-activating therapies in melanoma [171]. These efforts highlight the ongoing exploration of p53-targeted therapies in melanoma. There are ongoing clinical trials targeting p53 variants in melanoma treatment. One promising approach involves the use of MDM2 inhibitors such as navtemadlin, which aim to reactivate p53 in tumors with WT p53. Navtemadlin has shown potential in arresting melanoma growth and enhancing the effects of RT in preclinical models, and it is currently being tested in a phase II clinical trial [172].

DNA repair mechanisms play a crucial role in melanoma resistance to treatments and its development. The NER pathway is responsible for repairing UV-induced DNA damage. While NER is generally intact in melanoma, the global genome repair (GGR) branch is often reduced, leading to increased UV mutation loads [173]. Inhibition of DNA repair could make melanoma cells more vulnerable to DNA-damaging therapies, like RT. To inhibit DNA repair triggered by RT, Biau and colleagues developed an innovative class of molecules called Dbait (DNA strand break bait). These molecules are composed of 32 base pair deoxyribonucleotides that form an intramolecular DNA double helix, mimicking DNA damage. The model was tested on cell lines and mouse xenografts, demonstrating that DT01 (a clinical form of Dbait) enhanced RT efficacy independently of RT doses. Mice treated with a combination of DT01 and RT showed longer survival and improved tumor growth control without observing additional toxicity [174]. Moreover, a first-in-human phase I clinical trial was conducted to assess the safety, pharmacokinetics, and preliminary efficacy of intratumoral and peritumoral injections of DT01 in combination with RT for patients with unresectable melanoma skin metastases. It demonstrated that DT01 can be safely administered in combination with RT in patients with skin metastases from melanoma [175]. In another study, a small DNA molecule—coDbait in combination with arylcarboxamide derivatives (ICF01012) labeled with a β-emitting radionuclide iodine 131 ([^131^I] ICF01012)—was used to radiosensitize melanomas and increase targeted radionuclide therapy (TRT) efficacy. Such a combination significantly enhanced [^131^I]ICF01012 RT efficacy in human melanoma cells, inhibiting tumor growth and improving the survival in the syngeneic B16Bl6 model by disturbing DNA repair [176]. The MELRIV-1 trial (NCT03784625) is the first-in-human study evaluating [^131^I]ICF01012, a TRT, in patients with metastatic melanoma. This study aims to determine the recommended dose of [^131^I]ICF01012 for patients with pigmented metastatic melanoma [177].

## 5. Hypoxia and Altered Metabolism

In 1909, Gotwald Schwarz observed that reducing blood flow to irradiated skin decreased its radiation response. Subsequent research by Mottram, Crabtree, Cramer, and others extensively investigated the relationship between oxygen levels and radiation effects [178,179]. Nowadays, we know that hypoxic tumors are more resistant to RT due to a lower level of ROS causing DNA damage and due to the hypoxia-driven activation of the pro-survival and DNA repair signaling pathways [180,181]. Hypoxia refers to a condition where oxygen levels in tissues are below normal physiological levels, impairing cellular function and metabolism. In quantitative terms, hypoxia is typically defined as a partial pressure of oxygen (pO_2_) below 10 mmHg (compared to normal tissue pO_2_ levels of 40–60 mmHg) or an oxygen concentration below 1–2% O_2_ (compared to ~5% O_2_ in normal tissues) [181]. The epidermis naturally exists in a hypoxic state, with oxygen levels typically below 5% and sometimes reaching as low as 0.2%, particularly in the basal layers. This physiological hypoxia is a result of limited vascularization in the epidermis, as oxygen primarily diffuses from the dermis, which contains blood vessels. Oxygen levels decrease progressively from the dermis to the outermost layers of the epidermis. The basal layer, where keratinocytes proliferate, experiences the lowest oxygen levels, often below 1% [182]. Epidermal cells are adapted to low oxygen conditions. Hypoxia-inducible factors (HIFs) are constitutively active in keratinocytes, regulating various processes such as cell proliferation, differentiation, and barrier formation [88]. Melanoma cells, originating in the naturally hypoxic epidermis, are pre-adapted to survive and thrive under low oxygen conditions, which are further exacerbated in the tumor microenvironment. Therefore, physiological hypoxia is critical in the context of melanoma, as it influences tumor behavior and therapeutic resistance [183].

Melanoma is often defined by regions of hypoxia/anoxia resulting from an imbalance between oxygen consumption and delivery within the rapidly growing tumor mass [184]. The presence of hypoxia, namely measurement of oxygen tension pO_2_, is an independent prognostic factor for poor outcomes across various cancer types, including melanoma [185]. Hypoxia modifiers have been explored to enhance the effectiveness of RT since the early 1960s [186]. Oxygen mimetics, such as nitroimidazole derivatives, have been explored as hypoxia-targeting agents in melanoma treatment. These compounds mimic oxygen’s effects by radiosensitizing hypoxic tumor cells, which are typically resistant to RT. Nitroimidazole-based agents, like pimonidazole (PIMO) and etanidazole, selectively accumulate in hypoxic regions and enhance the efficacy of radiation by increasing DNA damage in oxygen-deprived melanoma cells. Additionally, these agents are being developed for dual purposes, including hypoxia imaging and therapeutic radiosensitization, offering a promising approach to overcoming hypoxia-induced therapy resistance in melanoma [187,188]. In the early 1980s, the radiosensitizing properties of electron-affinic nitroimidazoles, including metronidazole and misonidazole (Ro-07-0582), were evaluated in human malignant melanoma xenografts grown in athymic nude mice. Both compounds were administered intraperitoneally before irradiation with a 60Co therapy unit. The results showed that misonidazole was more effective than metronidazole and may be a more efficient hypoxic cell radiosensitizer for melanoma; thus, it could have potential value in human RT [189]. A subsequent study by Guichard and Malaise compared the radiosensitizing effects of misonidazole and a nitroimidazole analog, SR-2508, on a human melanoma (Na11) model that contained 85% hypoxic cells and was transplanted into nude mice. The results indicated that the difference in the radiosensitization enhancement ratios (RERs) between the two agents was related to the lack of potentially lethal damage (PLD) repair in tumors when the sensitizer was present. This suggests that both agents effectively radiosensitize hypoxic melanoma cells, but their mechanisms and efficacy may vary depending on their influence on DNA repair processes [190]. Gamoussi and Guichard investigated the uptake and radiosensitizing effects of PIMO and etanidazole (ETA) in vitro using two melanoma cell lines, namely Na11+ (pigmented) and Na11− (amelanotic). PIMO uptake was consistently higher than ETA in both cell lines and was slightly increased under hypoxic conditions compared to normoxia. The radiosensitizing effect of ETA was consistent across cell lines and growth phases. In contrast, the radiosensitizing effect of PIMO was cell line-dependent. The uptake and radiosensitizing effects of PIMO are influenced by melanin content, making this model useful for predicting the behavior of hypoxia-targeting agents in melanotic melanomas in vivo [191].

Hypoxia-inducible factor 1 (HIF-1) is a transcription factor activated by hypoxia, promoting radioresistance. Inhibiting HIF-1 can enhance the therapeutic effects of RT by reducing hypoxia-induced resistance. HIF1 is activated by the cAMP pathway and is a target of the MITF, which is crucial for melanocyte differentiation and melanoma progression. This activation leads to increased expression of vascular endothelial growth factor (VEGF), promoting angiogenesis and contributing to melanoma progression [192]. HIF1 influences the metabolic pathways in melanoma, such as glycolysis, enhancing the survival and invasiveness of cancer cells. This metabolic shift can contribute to drug resistance, making treatments less effective [144].

Targeting the hypoxic tumor microenvironment, which contributes to radioresistance, has been actively investigated for potential melanoma treatment. Inhibiting HIF-1 can enhance the therapeutic effects of RT by reducing hypoxia-induced resistance. For instance, inhibiting HIF1 can disrupt the tumor’s metabolic adaptation, making it more susceptible to cytotoxic therapies [193]. Acriflavine, a potent inhibitor of HIF-1α, has been studied for its ability to disturb glucose metabolism and suppress protective pathways in melanoma cells, even under normoxic conditions. This suggests a potential clinical application for targeting HIF-1α in melanoma, regardless of the tumor’s hypoxic status [194]. Research has shown that hypoxia can induce a switch in melanoma cells from a receptor tyrosine kinase-like orphan receptor 1 (ROR1)^+^ to a more invasive ROR2^+^ phenotype, which is associated with resistance to BRAF inhibitors. Another way to target hypoxia pathways is to target the Wnt5A/ROR2 axis that could improve the efficacy of treatments like vemurafenib in melanoma patients [195]. Targeting carbonic anhydrase XII (CAXII) has been shown to impair melanoma cell migration and invasion under hypoxic conditions. This strategy, whether through direct inhibition or modulation of the Hedgehog pathway, presents a potential novel therapeutic approach for melanoma treatment [196].

Hypoxia-activated prodrugs (HAP) are a promising approach for targeting hypoxic regions in melanoma, which are often resistant to conventional therapies. These prodrugs are selectively activated in low-oxygen environments, releasing cytotoxic agents that kill hypoxic tumor cells while sparing normal tissues [197]. A study by Zhang and Stevens investigated the effects of RT and the hypoxia-activated prodrug tirapazamine (SR-4233) on three melanoma cell lines (MM576, MM96L, and murine B16-F10). The oxygen enhancement ratios (OER) for single X-ray doses ranged between 2 and 3 for all cell lines, indicating significant hypoxia-induced radioresistance. However, adding tirapazamine to hypoxic cells before irradiation restored radiosensitivity to levels comparable to aerobic cells. This way, tirapazamine had little impact on the radiosensitivity of aerobic cells, highlighting its specificity for hypoxic conditions [198]. The combination of the hypoxia-activated prodrug TH-302 and sunitinib has been shown to enhance therapeutic effects in melanoma. Sunitinib, an anti-angiogenic agent, increases tumor hypoxia by inhibiting blood vessel formation. This hypoxic environment enhances the activation of TH-302, which selectively targets hypoxic tumor cells. In melanoma models, short-term sunitinib treatment alone failed to significantly prolong survival. However, when combined with TH-302, there was a marked increase in survival compared to either treatment alone. TH-302 effectively reduced tumor volume and targeted hypoxic compartments [199]. A phase I study evaluated the combination of evofosfamide (a hypoxia-activated prodrug) and ipilimumab (a CTLA-4 ICI) in advanced solid malignancies, including immunotherapy-resistant melanoma. Among 21 evaluable patients, 16.7% achieved partial responses, and 66.7% had stable disease. The best responses were observed at a dose of 560 mg/m^2^ evofosfamide. Improved T-cell proliferation and intratumoral T-cell infiltration were noted in responders [200].

Cell metabolism plays a crucial role in the radiation resistance of melanoma. Melanoma cells exhibit metabolic heterogeneity, allowing them to adapt and utilize various fuels for survival and progression, which can affect the efficacy of therapies, including RT. Key metabolic pathways, such as mitochondrial metabolism, are altered in melanoma and contribute to resistance against therapies. Melanoma exhibits metabolic plasticity, dynamically switching between glycolysis and oxidative phosphorylation (OXPHOS). This metabolic flexibility allows melanoma cells to adapt to challenging conditions and develop resistance to chemotherapy [201]. Changes in mitochondrial function can enhance cell survival and confer resistance to radiation [202]. Additionally, the tumor microenvironment influences metabolic adaptations, leading to altered glycolysis and other pathways that may promote tumorigenesis and resistance to treatment [203]. Recent research has revealed distinct metabolites and pathways, such as those involved in the metabolism of glycine, taurine, arginine, and alanine, that are altered in response to radiation. Exploring these metabolic changes could offer valuable insights into strategies for overcoming radiation resistance in melanoma [204]. Melanoma cells often shift from oxidative phosphorylation to aerobic glycolysis, which supports rapid ATP production and cell proliferation. This shift can enhance survival under stress conditions, including radiation [205].

Glutathione (GSH) and redox balance are critical factors influencing melanoma’s resistance to RT. High levels of GSH in melanoma cells can neutralize ROS, thereby reducing the effectiveness of RT [206]. Previous research has shown that ROS within the TME can induce an invasive phenotype in tumor-associated macrophages (TAMs) isolated from melanoma. Mechanistically, this effect may be attributed to the ROS-enhanced translocation of peroxisome proliferator-activated receptor γ (PPARγ) via the MAPK/ERK signaling pathway [207]. Targeting the GSH system or modulating redox balance may enhance the sensitivity of melanoma cells to radiation. For instance, using buthionine sulfoximine (BSO), an inhibitor of γ-glutamylcysteine synthetase (γGCS), which consequently lowers tissue GSH concentrations, increased apoptosis in melanoma cells, suggesting a potential strategy to overcome resistance [208]. This evidence clearly demonstrates that ROS plays a critical role in modulating the immune response within the TME of human melanoma, beyond its well-established role in oxidative stress. Overall, targeting metabolic pathways may enhance the effectiveness of RT therapy in melanoma treatment.

Radiosensitizers are emerging as valuable tools to enhance the efficacy of RT in melanoma by increasing tumor radiosensitivity while minimizing damage to surrounding healthy tissue. Key categories of melanoma radiosensitizers are presented in Table 1.

## 6. Activation of Anti-Tumor Immune Responses

RT induces DNA damage that not only reduces tumor burden but also enhances anti-tumor immunity. This process involves the activation of pathways, such as NF-κB, and type I interferon (IFN) responses, leading to the release of proinflammatory cytokines and the maturation of dendritic cells, which prime T-cell responses [211]. Melanoma is classified as an immunologically “hot” tumor, characterized by high levels of tumor-infiltrating lymphocytes (TILs), elevated expression of immune checkpoint molecules (e.g., PD-1, CTLA-4), and a high tumor mutational burden (TMB) [212]. These features make melanoma particularly responsive to ICIs, such as ipilimumab (anti-CTLA-4), nivolumab, and pembrolizumab (both anti-PD-1) [213]. ICIs have demonstrated durable responses in melanoma. For example, anti-PD-1 monotherapy achieves objective response rates (ORR) of ~40%, while combination therapy with anti-CTLA-4 increases ORR to ~58%, with long-term survival benefits [214]. Although ICIs have revolutionized melanoma treatment, many patients may not benefit due to therapy resistance. This resistance can be classified as primary (innate) or secondary (acquired). Innate ICI resistance in melanoma is associated with specific tumor subtypes characterized by invasive and dedifferentiated gene expression signatures. These tumors show reduced expression of melanocytic lineage markers (e.g., MITF) and increased expression of mesenchymal (e.g., AXL, ZEB1) and neural crest-like genes (e.g., SOX10, NGFR). These subtypes are associated with a low T cell-inflamed tumor microenvironment, characterized by reduced infiltration of CD8^+^ T cells and increased expression of immunosuppressive molecules, such as VEGF and TGF-β, which hinder immune activation [215,216]. Targeting inflamed (“hot”) and non-inflamed (“cold”) melanomas presents distinct biological and clinical challenges due to differences in their TME and immune profiles [217]. In the case of “hot” melanomas, resistance can develop due to adaptive immune suppression, such as the upregulation of alternative checkpoints (e.g., TIM-3, LAG-3) or recruitment of immunosuppressive cells, like regulatory T cells (Tregs) and myeloid-derived suppressor cells (MDSCs) [218]. “Cold” melanomas are resistant to ICIs due to the absence of pre-existing immune activation. These tumors lack TILs and exhibit poor antigen presentation, low neoantigen burden, and immunosuppressive TMEs driven by factors like TGF-β and VEGF. They are often associated with de-differentiated or mesenchymal-like gene expression profiles. Overcoming this requires strategies to convert “cold” tumors into “hot” ones [215,217].

The ongoing clinical trials highlight the efforts to optimize combination strategies for overcoming resistance and improving outcomes in melanoma treatment. One trial (NCT02843165) is investigating the combination of nivolumab and ipilimumab in patients with advanced melanoma. The study focuses on the efficacy of dual immune checkpoint blockade, aiming to improve PFS and OS compared to monotherapy. Early results suggest that the combination therapy shows promise in overcoming resistance mechanisms and enhancing anti-tumor immune responses.

The immune microenvironment plays a crucial role in the efficacy of RT. Certain factors, like tumor-associated macrophages (TAM) and T-cell infiltration, can significantly influence treatment outcomes, highlighting the importance of understanding these interactions for optimizing therapeutic strategies [219]. Gellert et al. investigated the role of tumor-derived IFNs in repolarizing TAMs in murine melanoma models and analyzed the impact of RT-induced type I IFN on TAMs in human melanoma patients. Their study showed that IFNβ induces a proinflammatory M1-like phenotype in TAMs in mice and identified a myeloid type I IFN-response signature linked to enhanced survival in melanoma patients undergoing RT. These findings suggest that type I IFN-inducing therapies, including RT, can effectively reprogram TAMs towards an anti-tumor phenotype, highlighting the potential of combining RT with immunotherapy to enhance treatment outcomes in melanoma [209].

Several clinical trials and studies have explored the combination of conventional RT and ICIs in melanoma. A systematic review and meta-analysis showed that combining RT with ICIs improved the ORR to 35%, compared to 20.39% with ICIs alone. PFS at 12 months was significantly better with the combination, although no clear OS advantage was observed [220]. The DeCOG multicenter retrospective cohort study investigated the impact of preceding RT on the outcomes of ICIs in metastatic melanoma. No significant differences in best overall response (BOR), PFS, or OS were observed between patients with and without preceding RT. In addition, in patients with brain metastases, preceding RT did not significantly improve survival outcomes when combined with ICI therapy [221]. The CHEERS phase II randomized clinical trial evaluated the combination of stereotactic body radiotherapy (SBRT) with ICIs in patients with advanced solid tumors, including melanoma. While safe, adding SBRT to ICIs did not significantly improve PFS or OS, highlighting the need for further research to optimize this combination [222].

Compared to conventional radio-immunotherapy, combining carbon ion RT (CIRT) with anti-PD-1 therapy more effectively induced immunogenic cell death (ICD) hallmarks, including calreticulin exposure, ATP release, and high mobility group box 1 (HMGB1) release, and stimulated type I IFN responses. This combination significantly increased CD4^+^ and CD8^+^ T-cell infiltration into tumors, leading to reduced tumor growth and prolonged survival in melanoma-bearing mice. These findings demonstrate that CIRT enhances tumor immunogenicity, amplifying the efficacy of subsequent anti-PD-1 immunotherapy [223]. Radiation stimulates dendritic cells, which are crucial for antigen presentation. This process helps prime T cells against tumor antigens, leading to a more robust anti-tumor immune response [66].

Li and colleagues demonstrated the synergistic anti-tumor effects of α-particle radionuclide therapy (α-TRT) combined with ICIs in preclinical melanoma models. The melanocortin 1 receptor (MC1R)-targeted radiopeptide [^212^Pb]VMT01 effectively suppressed the growth of B16-F10 melanoma tumors. Notably, the combination of [^212^Pb]VMT01 with ICIs led to a robust anti-tumor response, with 43% of mice achieving complete and durable tumor regression. This therapeutic synergy was dependent on T cell-mediated immunity and compromised by fractionated [^212^Pb]VMT01 administration. Mechanistically, [^212^Pb]VMT01 induced immunogenic cell death, sensitizing melanoma cells to ICI therapy and promoting the infiltration of CD3^+^, CD4^+^, and CD8^+^ T lymphocytes within the tumor microenvironment [210]. The first-in-human clinical trial evaluated the safety and biodistribution of two novel MC1R-targeted imaging tracers ([^203^Pb]VMT01 and [^68^Ga]VMT02) in stage IV melanoma patients to support the development of MC1R-targeted alpha-particle therapy. The study demonstrated tumor retention of tracers and partial concordance between imaging and immunohistochemistry (IHC), suggesting that IHC is a more sensitive method for detecting MC1R expression, with further testing needed to refine imaging-based patient selection for therapy [224].

A study by Yu et al. demonstrated that melanoma liver metastases act as an immune sink, trapping and eliminating circulating activated CD8^+^ T cells in multiple mouse models. In the liver, activated antigen-specific Fas^+^CD8^+^ T cells are selectively induced to undergo apoptosis by FasL^+^CD11b^+^F4/80^+^ monocyte-derived macrophages. This phenomenon contributes to a systemic immunosuppressive state, characterized by reduced peripheral T-cell numbers and diminished tumor-infiltrating T-cell diversity and function in both preclinical models and human patients with melanoma liver metastases. Importantly, liver-directed RT effectively eliminates immunosuppressive hepatic macrophages in preclinical models, enhancing hepatic T-cell survival and mitigating the detrimental effects of hepatic T-cell siphoning [225]. These findings suggest that irradiation of liver metastases can enhance the effectiveness of ICI therapies. This highlights the crucial role of timing and the importance of considering liver-metastasis-directed radiotherapy (LRT) as a potential strategy to improve ICI response [226]. A recent study by Jagodinsky and co-authors investigated the temporal dynamics of IFN1 activation following both external beam radiation therapy (EBRT) and TRT across various tumor models, including melanoma. The authors demonstrated that TRT effectively induces IFN1 activation comparable to EBRT, highlighting its potential for synergistic combination with immunotherapies [227]. Localized RT can lead to systemic anti-tumor effects, known as the abscopal effect, where non-irradiated tumors shrink due to immune activation. This immune-mediated effect is crucial for the abscopal response, which can be augmented by combining radiation with immunotherapeutic agents, like ICIs [228,229].

## 7. Pigmentation and Melanin Production

Melanoma pigmentation plays a significant role in radiation sensitivity and resistance. Melanin, particularly eumelanin, provides radioprotection by acting as an antioxidant and reducing DNA damage from UV radiation. A study by Meredith and Sarnan investigated the electronic and optical properties of four monomers that serve as fundamental building blocks of eumelanin. Using Density Functional Theory (DFT) and Time-Dependent DFT (TDDFT) calculations, the authors determined key molecular properties, including electron affinities, ionization energies, energy gaps, optical absorption spectra, and exciton binding energies, and discussed their implications for the properties of a proposed tetrameric protomolecule of eumelanin [230].

In contrast, pheomelanin, which is less stable, can generate a mutagenic environment under UV exposure, potentially contributing to melanoma progression and resistance to therapies, including RT [231]. Furthermore, radiation can stimulate melanocytes, leading to increased melanin production and subsequent hyperpigmentation [232]. The MC1R genotype influences the response of melanocytes to UV radiation. Variants associated with red hair and fair skin reduce the protective effects of melanin, increasing sensitivity to UV-induced damage. MC1R allelic variants often exhibit impaired cAMP signaling, leading to a shift towards pheomelanin production and enhanced extracellular signal-regulated kinase (ERK) pathway activation. This altered pigmentation profile, characterized by increased pheomelanin content, may contribute to the increased melanoma risk associated with these variants, suggesting that modulation of this pathway could improve treatment outcomes [233].

Moreover, the degree of pigmentation in melanoma cells correlates with their radiosensitivity; higher eumelanin content is associated with increased resistance to radiation [234,235]. DOPAchrome tautomerase TRP-2, an enzyme crucial for eumelanin synthesis, is significantly upregulated in radioresistant WM35 melanoma cells, likely due to overexpression of the ERK/MAPK pathway [236]. Kinnaert and colleagues conducted a study examining the relationship between cell pigmentation and radiosensitivity in two human melanoma cell lines with differing melanin content (mixed and pheomelanotic). Their findings revealed an inverse correlation between eumelanin levels and radiosensitivity. Increased melanin levels, regardless of the inducing factor, promoted the growth of irradiated cells. These findings suggest that eumelanin content may significantly influence the radiosensitivity of melanoma cells, potentially contributing to the variability observed in clinical responses to RT [235]. In another study, the authors showed that higher intracellular eumelanin levels are inversely associated with DNA damage, even in the absence of glutathione and cysteine, two essential intracellular radioprotective agents. These findings suggest that enhancing eumelanin production, either through tyrosine supplementation or by shifting melanin synthesis towards eumelanin, can compensate for the loss of these crucial radioprotective molecules [237]. Normalizing tumor vasculature and reducing hypoxia can enhance the effectiveness of RT, indicating that strategies to inhibit eumelanogenesis might synergize with other therapeutic approaches to improve overall treatment outcomes [238].

**Figure 1:**
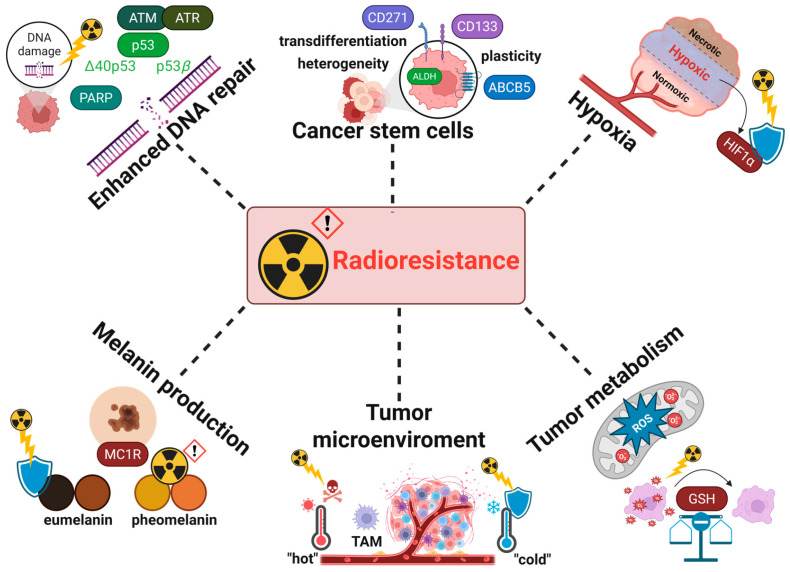
Factors contributing to radioresistance in melanoma. These include enhanced DNA repair mechanisms (involving, e.g., PARP, ATM, ATR, and p53), the presence and characteristics of cancer stem cells (CSCs) exhibiting plasticity, heterogeneity, and transdifferentiation (marked by surface markers, such as CD271, CD133, ABCB5, and ALDH), hypoxia-induced resistance (mediated by HIF1α), altered tumor metabolism (involving ROS and GSH), melanin production (with MC1R signaling), and the complex tumor microenvironment (TME) where melanoma can be classified as “hot” or “cold”, with tumor-associated macrophages (TAMs) contributing to RT resistance. The interplay of these factors governs the overall resistance of melanoma cells to radiation therapy. Created in https://BioRender.com.

In summary, enhanced DNA repair capabilities allow melanoma cells to efficiently recover from radiation-induced damage, particularly through the overexpression of repair genes associated with stalled replication forks. The TME plays a protective role by fostering immune evasion, promoting angiogenesis, and shielding tumor cells from radiation effects. Pigmentation and melanin production further contribute by absorbing radiation and scavenging reactive oxygen species, reducing DNA damage. Hypoxia within the tumor amplifies radioresistance by reducing oxidative stress and stabilizing HIFs, which promote survival pathways and reduce radiation efficacy. Altered tumor metabolism, including increased glycolysis and oxidative stress adaptation, supports energy demands and resistance mechanisms. Additionally, CSCs within melanoma exhibit intrinsic radioresistance due to their quiescent nature, efficient DNA repair, and ability to repopulate the tumor post-treatment. Together, these factors create a robust resistance network, complicating RT effectiveness and necessitating targeted strategies to overcome these barriers (Figure 1).

## 8. Combination of Radiotherapy with Other Anti-Cancer Treatments

Combining RT with immunotherapy, such as ICIs, has shown potential for improved outcomes. Studies indicate that this combination can enhance T-cell infiltration and OS in melanoma patients, suggesting a synergistic effect [239,240]. Specific biomarkers have been identified that may predict the effectiveness of RT combined with immunotherapy in melanoma. The combination of fibroblast activation protein (FAP)-targeted molecular RT with immunotherapy has shown promising results in preclinical models, suggesting that this approach can lead to tumor regression and increased apoptotic cell death, which may also be influenced by specific immune cell populations [241]. Shi and colleagues applied a multi-omics approach and highlighted the role of RT-related genes, such as dual specificity phosphatase 1 (DUSP1), C-X-C motif chemokine ligand 13 (CXCL13), SLAM family member 7 (SLAMF7), and ecotropic viral integration site 2B (EVI2B). These genes were shown to boost immune responses and enhance the effectiveness of immunotherapy in melanoma patients, suggesting their potential as predictive biomarkers for treatment success [56].

There are also ongoing clinical trials investigating the combination of RT and immunotherapy in melanoma. One phase I study evaluated the safety and immunologic effects of combining RT with nivolumab and ipilimumab in patients with stage IV melanoma. The study concluded that RT combined with nivolumab and ipilimumab is safe and may enhance immunologic effects, with ongoing randomized trials to assess efficacy [242]. The NADINA trial (NCT04949113) is currently evaluating the efficacy of neoadjuvant systemic therapy combining ipilimumab and nivolumab followed by surgery, compared to standard adjuvant therapy. This phase III trial demonstrates that neoadjuvant treatment with ipilimumab and nivolumab is safe and results in improved event-free survival compared to adjuvant nivolumab in patients with resectable macroscopic stage III melanoma [243]. A retrospective study of 376 patients with melanoma brain metastases (MBMs) treated with ipilimumab plus nivolumab (COMBO) demonstrated remarkable long-term survival in treatment-naïve, asymptomatic, steroid-free patients, with median overall survival (mOS) not yet reached in this subgroup. The addition of stereotactic radiosurgery (SRS) to COMBO significantly improved survival, while outcomes were poor in patients receiving COMBO after progression on BRAF/MEK inhibitors [244]. The ReCIPE-M1 (NCT04581382) study investigated the safety and feasibility of therapeutic plasma exchange (TPE) to improve outcomes in metastatic melanoma patients progressing through PD-L1 immunotherapy. By removing extracellular vesicles (ev) expressing PD-L1 and soluble (s) PD-L1, TPE is hypothesized to restore anti-melanoma immunity, with primary endpoints focused on safety and secondary endpoints evaluating sPD-L1 kinetics and clinical response [245]. Next, the authors presented a phase I clinical trial examining the combination of RT, TPE, and ICI rechallenge in patients with ICI-refractory metastatic melanoma exhibiting high PD-L1 levels. This study provides clinical evidence that combining limited SBRT, TPE, and ICI rechallenge may reinstate responsiveness to immune checkpoint inhibitors [246]. Ongoing studies are examining the potential of RT to induce abscopal effects, where localized treatment leads to systemic anti-tumor responses, particularly when combined with immunotherapy [247,248].

A study by Bonnen et al. demonstrated the efficacy and safety of elective regional RT (the use of RT to treat areas where cancer cells are likely to spread, even if there is no evidence of cancer in those areas yet) for head and neck melanoma patients at high risk of lymph node involvement. It provides a viable alternative to elective lymph node dissection [249]. A study by Fogarty revealed that adjuvant radiation therapy (ART) utilizing three-dimensional conformal radiotherapy (3DCRT) reduced in-field recurrence by 50% in patients with macroscopic regional nodal melanoma. This approach is now being evaluated in combination with targeted therapies and immunotherapy [250].

EBRT is increasingly utilized in the treatment of melanoma, particularly in combination with other therapies. EBRT has been shown to activate a type 1 interferon (IFN1) response in melanoma cells, which is crucial for enhancing the effectiveness of immunotherapies such as ICI. This activation occurs within days post-radiation and can persist, suggesting a potential synergistic effect when combined with immunotherapy [227]. Studies indicate that combining EBRT with targeted radionuclide therapies (TRTs) can enhance tumor uptake of therapeutic agents and improve treatment efficacy. For instance, EBRT combined with TRT in melanoma models demonstrated significant tumor growth inhibition compared to monotherapies [251]. EBRT is often utilized in the treatment of cutaneous melanoma, particularly in cases of metastatic disease. A study by Greene and co-authors indicated that EBRT was used in 47% of cases with orbital metastasis from cutaneous melanoma, showing varying degrees of tumor control depending on the treatment combination employed [252]. The integration of EBRT with immunotherapy and targeted therapies is a promising area of research, aiming to overcome treatment resistance and improve patient outcomes [253].

An alternative strategy involves the use of stereotactic radiosurgery (SRS) to treat brain metastases from melanoma, offering effective local tumor control and potential survival advantages. Liew et al. performed a study involving 333 consecutive patients with melanoma and showed that SRS achieved a local control rate of 73% and a median survival of 22 months for those with solitary brain metastases and controlled extracranial disease [254]. Another analysis indicated that SRS combined with immunotherapy, such as ipilimumab, significantly improved survival rates compared to SRS alone, with a hazard ratio of 0.74, suggesting a synergistic effect [255]. However, patients with melanoma brain metastases generally have poorer outcomes, with a median survival of approximately 7.5 months post-SRS. Factors influencing survival include the number of brain metastases, prior treatments, and the control of systemic disease [256]. Overall, SRS is a valuable option for managing melanoma brain metastases, especially when combined with systemic therapies, although careful patient selection is crucial for optimizing outcomes.

Boron neutron capture therapy (BNCT) is an innovative treatment approach for cutaneous melanoma that utilizes the selective accumulation of boron-10 in tumor cells, followed by irradiation with thermal neutrons. This process generates high-energy alpha particles that selectively destroy cancer cells while sparing surrounding healthy tissue [239]. Clinical studies have demonstrated the potential of BNCT in treating melanoma, particularly in patients who have not responded to conventional therapies. For instance, the use of boronophenylalanine (BPA) has shown promise in preclinical models, where it effectively targeted melanoma cells, leading to significant tumor control [257]. A phase I/II clinical trial is assessing the safety and efficacy of BNCT for treating malignant melanoma. Initial results from the first treated patient showed promising outcomes with manageable side effects [258]. However, challenges remain regarding the optimal delivery of boron to tumor sites and the precise measurement of boron concentration in tissues, which are critical for maximizing treatment efficacy [259].

Proton beam therapy (PBT) is another advanced form of RT that uses protons to target tumors, including cutaneous melanoma. While PBT is more commonly associated with non-melanoma skin cancers, its application in melanoma is being explored. Ongoing studies are investigating the optimal use of PBT in melanoma treatment, including its combination with immunotherapy to enhance systemic responses [260]. One study examined the outcomes of patients with head and neck cutaneous melanoma treated with PBT by utilizing data from a multi-institutional registry. Findings indicate that PBT effectively controls local disease in patients with locally advanced melanoma, predominantly in the adjuvant setting. Three-year local regional failure-free survival (LRFS), PFS, and OS rates were 85.7%, 35.7%, and 83.3%, respectively [261].

The single-center, first-in-human, phase I study IMPulse (NCT04986696) was initiated to investigate the safety and efficacy of FLASH therapy in patients with metastatic melanoma. Utilizing Mobetron^®^ with high-dose-rate (HDR) functionality, the study employed a dose-escalation approach to evaluate single-dose FLASH therapy for skin metastases that progressed despite systemic treatments. A study by Dong et al. combined FLASH-RT with imiquimod (IMQ), a Toll-like receptor (TLR) agonist, delivered via a radiopaque and radiation-responsive hydrogel. The combination therapy significantly reduced tumor growth and improved survival in preclinical models with enhanced cytokine levels in the tumor microenvironment, indicating immune activation. The hydrogel was radiopaque, allowing for monitoring via CT imaging, and no adverse effects were reported [52]. Hypofractionated RT, particularly when used alongside anti-PD-1 therapy, has demonstrated increased efficacy in advanced melanoma patients, leading to higher rates of complete responses [262]. Studies have shown that combining RT with BRAF/MEK inhibitors can lead to improved outcomes, especially in patients who have developed resistance to these targeted therapies. One case report highlighted the successful treatment of BRAF/MEK inhibitor-resistant melanoma using a combination of immunotherapy followed by intensity-modulated radiotherapy (IMRT), which resulted in significant tumor regression [263]. In BRAF-mutated melanoma, combining radiotherapy with BRAF inhibitors (e.g., vemurafenib) and p53 reactivation has been shown to enhance radiosensitivity, potentially overcoming the inherent radioresistance of melanoma cells [264].

MRT using spatially fractionated high-dose microbeams has demonstrated significant tumor control in preclinical melanoma models. For example, temporally fractionated MRT with lower peak doses achieved complete remission in 50% of B16-F10 mouse melanomas, preventing metastases and increasing survival [265]. MRT also shows potential to overcome melanoma radioresistance by enhancing local and systemic anti-tumor immune responses [53].

Palliative radiotherapy (PRT) is an important treatment option for patients with cutaneous melanoma, particularly in advanced stages where curative treatments are no longer effective. PRT for melanoma patients primarily alleviates several key symptoms associated with the disease. A significant number of patients experience pain due to tumor growth, particularly in cases with bone metastases. PRT can provide effective pain relief, with studies showing that up to 67% of patients with bone metastases report good pain management [266]. For patients with brain metastases, PRT can ameliorate neurological deficits, with studies indicating that around 57% of patients experience improvements in their neurological function [266]. PRT can also help manage ulceration and other skin-related symptoms, contributing to better overall QoL [65]. PRT is effective in managing bleeding from tumors, especially in cases where surgical options are limited. It can help reduce or stop hemorrhaging, improving patient comfort [54].

In cases where patients have previously failed systemic therapies, such as anti-PD-1 monotherapy, combining PRT with these treatments has demonstrated promising results. For instance, a study by Saiag et al. indicated that hypofractionated RT combined with anti-PD-1 therapy led to high rates of complete response and improved OS compared to those who did not receive RT [262]. The integration of PRT with immunotherapy represents a valuable strategy in the management of melanoma, potentially leading to better therapeutic outcomes [267,268].

Potential biomarkers can help predict which melanoma tumors are most likely to benefit from combined RT and systemic strategies, such as ICIs or radiosensitizers. Exemplary biomarkers include PD-L1 expression, TMB, and immune infiltration profiles. High PD-L1 expression on melanoma cells or in the tumor microenvironment may predict better responses to combined RT and ICIs, as RT can enhance PD-L1-mediated immune activation [55]. High TMB is associated with increased neoantigen presentation, which may enhance the efficacy of RT-ICI combinations by promoting immune recognition [269]. Tumors with pre-existing immune infiltration (e.g., high CD8^+^ T-cell density) are more likely to benefit from RT-ICI combinations, as RT can further enhance immune activation [56].

Combining RT with other agents enhances therapeutic efficacy by targeting multiple pathways, overcoming resistance, and improving immune responses (Table 2).

## 9. Future Perspectives

The number of papers pointing out the need for new therapeutic strategies for melanoma patients has grown vastly during the last decade. Future perspectives of melanoma radiation resistance research should focus on overcoming the intrinsic and acquired resistance mechanisms through innovative strategies. From the biological point of view, improved preclinical models, such as genetically engineered mouse models and patient-derived models in humanized mice, are being developed to better predict clinical responses to RT and other therapies [270,271]. Advanced in vitro models, such as 3D reconstructed models, capillary network formation models, and organoids, are increasingly used in melanoma research to better mimic the TME and improve translational relevance [271]. Advances in understanding the molecular mechanisms of melanoma, including mutations in BRAF, NRAS, and c-KIT, are guiding the development of targeted therapies that may complement RT.

Combining RT with immunotherapy, such as ICIs, is being explored to overcome resistance and enhance treatment efficacy [272]. However, high tumor burden, hypoxic and acidic tumor microenvironment, and stromal barriers can limit the effectiveness of both radiation and immunotherapy. Overcoming these barriers remains a significant challenge. Variability in patient responses to combined therapies complicates treatment optimization. Reliable biomarkers to predict individual sensitivity and efficacy are still lacking.

Oncolytic virus (OV) therapy, either alone or in combination with RT, is showing potential for reducing recurrence rates and improving outcomes in melanoma patients [273]. OVs exploit the altered antiviral pathways in melanoma cells, such as defective interferon signaling, allowing the virus to replicate preferentially in tumor cells while sparing normal cells. Melanoma cells often overexpress specific receptors that OVs can bind to, e.g., ICAM-1 [274] and Nectin-1 [275], enhancing their ability to infect melanoma cells selectively [276].

CTCs play a critical role in melanoma radioresistance and metastatic progression. Recent studies highlight the “Anti-Warburg Effect” (AWE) in CTCs, where a metabolic shift from glycolysis to oxidative phosphorylation occurs, potentially bridging primary tumors and metastases. This metabolic adaptation may enhance CTC survival under therapeutic stress, including radiation, and is significantly correlated with therapeutic response in melanoma patients [277]. Although the current paper may not address CTC-mediated radioresistance, future discussions could explore how targeting the AWE in CTCs might improve therapeutic outcomes. This could include strategies to disrupt metabolic plasticity or combine RT with metabolic inhibitors to prevent metastasis and overcome resistance.

Emerging data suggest that mRNA vaccines could enhance immune responses when combined with RT, offering a novel therapeutic pathway [278]. These vaccines encode tumor-specific antigens, enhancing the immune system’s ability to recognize and attack melanoma cells. They can induce both humoral and cellular immune responses, and their rapid production makes them adaptable for personalized therapies [279]. Radiation and mRNA vaccines together increase the infiltration of CD8^+^ T cells and cytokine production, enhancing anti-tumor immunity [280]. RT can enhance the immunogenicity of tumors by releasing tumor antigens, which mRNA vaccines can further exploit to stimulate a robust immune response. This synergy may improve tumor control and reduce recurrence rates [281]. A pilot study using mRNA-electroporated dendritic cells (TriMix-DC) encoding melanoma antigens demonstrated safety and immunogenicity. While no objective responses were observed with the vaccine alone, combining it with interferon-alpha-2b showed partial responses and stable disease in some patients [282]. A phase I trial of the ECI-006 vaccine (TriMix-based mRNA) in resected melanoma patients showed that it was well-tolerated and immunogenic. Further studies are planned to combine it with anti-PD-1 therapy [283]. The KEYNOTE-942 phase II trial evaluated the mRNA-4157 vaccine combined with pembrolizumab in high-risk melanoma patients. The combination significantly improved recurrence-free survival (RFS) and distant metastasis-free survival (DMFS) compared to pembrolizumab alone. The risk of recurrence or death was reduced by 44%, and the 18-month DMFS rate was 91.8% for the combination versus 76.8% for monotherapy [284,285]. The combination of the mRNA vaccine approach with RT and other treatment modalities warrants further investigation. Moreover, further preclinical and clinical studies are needed to determine the optimal timing, dose, and fractionation of RT when combined with other treatments, such as checkpoint inhibitors or mRNA vaccines, to maximize synergy and minimize toxicity.

## 10. Conclusions

This review provides a comprehensive overview of the mechanisms driving melanoma radioresistance, including enhanced DNA repair capabilities, the protective role of the TME, and the influence of melanin production. These factors collectively contribute to the limited efficacy of RT in melanoma treatment. However, the integration of novel therapeutic strategies, such as ICIs, targeted radionuclide therapies, and DNA repair inhibitors, offers promising avenues to overcome these challenges. Preclinical and clinical evidence suggest that combining RT with systemic therapies, including BRAF/MEK inhibitors and ICIs, can synergistically enhance anti-tumor immune responses and improve treatment outcomes.

Ongoing and planned clinical trials are actively addressing radioresistance in melanoma by testing radiosensitizers and combined RT-immunotherapy strategies. For example, studies are exploring the use of nanoparticle-based radiosensitizers to enhance RT efficacy and synergize with ICIs to elicit systemic anti-tumor immunity, including abscopal effects [286,287]. Additionally, trials combining RT with a dual checkpoint blockade (e.g., anti-CTLA4 and anti-PD-1/PD-L1) have shown promise in overcoming resistance by modulating the tumor microenvironment and reversing T-cell exhaustion [269].

Future research should focus on optimizing these combination strategies and exploring advanced RT techniques, such as FLASH therapy and hypofractionated RT, to further enhance therapeutic efficacy. Promising avenues include the integration of radiosensitizers, such as nanoparticle-based agents, to improve RT outcomes, and the combination of RT with ICIs to exploit synergistic effects and induce systemic anti-tumor immunity. Additionally, targeting hypoxia-induced resistance and metabolic vulnerabilities such as glutathione pathways represents a promising direction for sensitizing melanoma cells to RT. By addressing the multifaceted mechanisms of radioresistance and leveraging innovative therapeutic approaches, progress can be made in improving survival and quality of life for melanoma patients. This underscores the importance of continued translational research to bridge the gap between mechanistic insights and clinical application.

All in all, cancer is increasingly recognized as a complex, adaptive system rather than merely a collection of individual cells with altered molecular pathways. This perspective emphasizes the dynamic interactions among cancer cells, their microenvironment, and systemic factors, which collectively drive tumor progression, resistance, and metastasis. Viewing resistance as a property of a complex system suggests that targeting single molecular pathways may be insufficient. Instead, adaptive, multi-targeted, and dynamic treatment strategies are needed to disrupt the system’s resilience.

## Figures and Tables

**Table 1:** Selected melanoma radiosensitizers.

Modulator/Agent	Mechanism of Action	Stage of Research	References
Peposertib	DNA-PKcs	Preclinical	[148]
CC-115	mTOR, DNA-PK	Preclinical	[149]
Olaparib	PARP-1	Clinical	[150,151,152]
AZD-6738	PARP-1	Preclinical	[152]
Prexasertib	CHK1	Preclinical	[153]
Sodium butyrate	HDAC	Preclinical	[154]
Ceralasertib	ATR	Clinical Phase II	[155]
Durvalumab	PD-L1	Clinical Phase II	[155]
Navtemadlin	MDM2/p53	Preclinical/Clinical Phase II	[172]
DT01	Dbait	Preclinical/Clinical Phase I	[174,175]
[^131^I]ICF01012	coDbait	Preclinical/Clinical Phase I	[176,177]
Misonidazole	Hypoxia	Preclinical	[189]
Etanidazole	Hypoxia	Preclinical	[191]
Acriflavine	HIF-1α	Preclinical	[194]
Tirapazamine	Hypoxia	Preclinical	[198]
TH-302	Hypoxia	Preclinical	[199]
Evofosfamide	Hypoxia	Preclinical/Clinical Phase I	[200]
Buthionine sulfoximine	γGCS	Preclinical	[208]
Type I IFN Inducers	Immune	Preclinical	[209]
[^212^Pb]VMT01	MC1R-targeted radiopeptide	Preclinical	[210]

**Table 2:** Selected recent clinical trials involving RT for melanoma.

Name	Type of Trial	Disease	Radiotherapy Scheme	Systemic Treatment	Patients	Reference
Published Trials						
A Prospective, Phase I Trial of Nivolumab, Ipilimumab, and Radiotherapy in Patients with Advanced Melanoma	Phase I, non-randomized	Metastatic melanoma	30 Gy, 10 fractions 27 Gy, 3 fractions	Nivolumab, ipilimumab	20 patients	[242]
Phase I Study of [^131^I] ICF01012, a Targeted Radionuclide Therapy (MELRIV-1)	An open-label, multicentric, dose-escalation phase I trial	Pigmented metastatic melanoma	Targeted radionuclide therapy, escalating doses of a radiolabeled compound [^131^I]ICF01012		36 patients	[177]
The CHEERS Phase II Randomized Clinical Trial	An open-label, multicenter, randomized phase II trial	Locally advanced or metastatic melanoma	SBRT, 8 Gy, 3 fractions	ICI: Nivolumab, pembrolizumab, atezolizumab	24 patients	[222]
Targeted Imaging of Melanoma for Alpha-Particle Radiotherapy (TIMAR1)	Phase I	Advanced melanoma	[^203^Pb]VMT01 [^68^Ga]VMT02		7 patients	[224]
Combined Immunotherapy in Melanoma Patients with Brain Metastases: A Multicenter International Study	Not specified	Melanoma brain metastases	Not specified	Ipilimumab, nivolumab	376 patients	[244]
Radiation Therapy, Plasma Exchange, and Immunotherapy (Pembrolizumab or Nivolumab) for the Treatment of Melanoma	Phase I	Melanoma	40 Gy, 5 fractions	Nivolumab, pembrolizumab	18 patients	[245,246]
Planned Trials (with unpublished results)						
Anti-PD 1 Brain Collaboration + Radiotherapy Extension (ABC-X Study) (ABC-X)	Phase II, randomized	Melanoma brain metastases	Stereotactic radiotherapy from 16 to 22 Gy in 1 fraction or from 24 to 30 Gy, hypofractionated for larger lesions	Ipilimumab, nivolumab	218 patients	NCT03340129 Start 08/2019
Precision Radiation of Immune Checkpoint Therapy Resistant Melanoma Metastases (PROMMEL)	Phase II	Immune checkpoint-resistant melanoma metastases	Not specified	PD-1 inhibitor	27 patients	NCT04793737 Start 03/2021
Irradiation of Melanoma in a Pulse (IMPulse)	Phase I, non-randomized	Skin metastases of melanoma	7 dose levels (22 Gy, 24 Gy, 26 Gy, 28 Gy, 30 Gy, 32 Gy, and 34 Gy)	None	46 patients	NCT04986696 Start 07/2021
Nodal Radiation Therapy for Sentinel Lymph Node Positive Melanoma (MelPORT)	Phase II, randomized	Sentinel lymph node-positive melanoma	30 Gy, 5 fractions	Nivolumab, pembrolizumab	168 patients	NCT04594187 Start 08/2021
PD-1 Inhibitors with or without Radiation in Advanced Melanoma	Phase II, randomized	Advanced melanoma	Not specified	PD-1 inhibitor	92 patients	NCT05498805 Start 08/2022
Stereotactic Ablative Radiotherapy (XRT) and Immunotherapy for Oligometastatic Extracranial Melanoma (AXIOM)	Phase II, randomized	Metastatic melanoma	48 Gy, 10 fractions	Immunotherapy	129 patients	NCT06767306 Start 04/2025

## Abbreviations

The following abbreviations are used in this manuscript:

α-TRT	Alpha-particle radionuclide therapy
ABC	ATP-binding cassette
ABCB5	ATP-binding cassette sub-family B member 5
ALDH	Aldehyde dehydrogenase
AM	Acral melanoma
ART	Adjuvant radiation therapy
ATM	Ataxia telangiectasia-mutated
ATP	Adenosine triphosphate
BPA	Boronophenylalanine
BRAF	B-Raf Proto-oncogene
BNCT	Boron neutron capture therapy
bsAbs	Bispecific antibodies
cAMP	Cyclic adenosine monophosphate
CAR-T	Chimeric antigen receptor T cell
CD3^+^	Cluster of differentiation 3 positive
CD4^+^	Cluster of differentiation 4 positive
CD8^+^	Cluster of differentiation 8 positive
CHK1	Checkpoint kinase 1
CIRT	Carbon ion RT
CM	Cutaneous melanoma
CSCs	Cancer stem cells
CTCs	Circulating tumor cells
CXCL13	C-X-C motif chemokine ligand 13
Dbait	DNA strand break bait
DDRs	DNA damage responses
DOPA	Dihydroxyphenylalanine
DSBs	DNA double-strand breaks
DT01	Clinical form of Dbait
DUSP1	Dual specificity phosphatase 1
EBRT	External beam radiation therapy
ECM	Extracellular matrix
ERK	Extracellular signal-regulated kinase
EVI2B	Ecotropic viral integration Site 2B
FAP	Fibroblast activation protein
FLASH	Ultra-high-dose rate radiation therapy
HAP	Hypoxia-activated prodrug
HDAC	Histone deacetylase
HDR	High-dose-rate
HIF-1	Hypoxia-inducible factor 1
HMGB1	High mobility group box 1
ICB	Immune checkpoint blockade
ICF01012	Arylcarboxamide derivative labeled with iodine-131
ICIs	Immune checkpoint inhibitors
IFN1	Type 1 interferon
IFNβ	Interferon beta
IMRT	Intensity-modulated radiotherapy
IMQ	Imiquimod
KIT	KIT proto-oncogene
LINAC	Linear accelerator
LRT	Liver-metastasis-directed radiotherapy
LRFS	Local regional failure-free survival
MC1R	Melanocortin 1 receptor
MITF	Microphthalmia-associated transcription factor
MM	Mucosal melanoma
NF-κB	Nuclear factor kappa B
NF1	Neurofibromatosis type I
NRAS	Neuroblastoma RAS viral oncogene homolog
OER	Oxygen enhancement ratio
OS	Overall survival
p53	Protein p53
PBT	Proton beam therapy
PD-1	Programmed cell death protein 1
PFS	Progression-free survival
PIMO	Pimonidazole
PO2	Oxygen tension
PRT	Palliative radiotherapy
RER	Radiosensitization enhancement ratio
RT	Radiation therapy
SLAMF7	SLAM family member 7
TAMs	Tumor-associated macrophages
TIL	Tumor-infiltrating lymphocyte
TLR	Toll-like receptor
TMB	Tumor mutational burden
TRP-2	Tyrosinase-related protein 2
TRT	Targeted radionuclide therapy
QoL	Quality of life

## References

[1] Heistein J.B., Acharya U., Mukkamalla S.K.R. (2025). Malignant Melanoma. StatPearls.

[2] Lin W., Wang W., Hodi F.S., Min L. (2025). Gaps in the Management of Adrenal Insufficiency in Melanoma Survivors: A Retrospective Cohort Study. eClinicalMedicine.

[3] Manikandan S.P., Narani S.R., Karthikeyan S., Mohankumar N. (2025). Deep Learning for Skin Melanoma Classification Using Dermoscopic Images in Different Color Spaces. Int. J. Electr. Comput. Eng. IJECE.

[4] Zheng S., Yu H., Zheng X., Wu U.T., Ming W., Huang H., Song J., Zhang X., Lyu J., Deng L. (2023). Analysis and Prediction of 5-Year Survival in Patients with Cutaneous Melanoma: A Model-Based Period Analysis. Front. Endocrinol..

[5] Rashed H., Flatman K., Bamford M., Teo K.W., Saldanha G. (2017). Breslow Density Is a Novel Prognostic Feature in Cutaneous Malignant Melanoma. Histopathology.

[6] Lo S.N., Williams G.J., Cust A.E., Varey A.H.R., Ch’ng S., Scolyer R.A., Thompson J.F. (2025). Long-Term Survival across Breslow Thickness Categories: Findings from a Population-Based Study of 210042 Australian Melanoma Patients. JNCI J. Natl. Cancer Inst..

[7] Brewer J.D., Christenson L.J., Weaver A.L., Dapprich D.C., Weenig R.H., Lim K.K., Walsh J.S., Otley C.C., Cherikh W., Buell J.F. (2011). Malignant Melanoma in Solid Transplant Recipients: Collection of Database Cases and Comparison with Surveillance, Epidemiology, and End Results Data for Outcome Analysis. Arch. Dermatol..

[8] Chang S.T., Desser T.S., Gayer G., Menias C.O. (2014). Metastatic Melanoma in the Chest and Abdomen: The Great Radiologic Imitator. Semin. Ultrasound CT MRI.

[9] Gener Lahav T., Adler O., Zait Y., Shani O., Amer M., Doron H., Abramovitz L., Yofe I., Cohen N., Erez N. (2019). Melanoma-Derived Extracellular Vesicles Instigate Proinflammatory Signaling in the Metastatic Microenvironment. Int. J. Cancer.

[10] Suman S., Markovic S.N. (2023). Melanoma-Derived Mediators Can Foster the Pre-Metastatic Niche: Crossroad to Lymphatic Metastasis. Trends Immunol..

[11] Khan R.R., Kahlon S., Maragh J.-P., Kimble A., Gupta S., Khan R.R., Kahlon S., Maragh J.-P., Kimble A., Gupta S. (2024). A Rare Case of Metastatic Adrenal Melanoma. Cureus.

[12] Rhodin K.E., Fimbres D.P., Burner D.N., Hollander S., O’Connor M.H., Beasley G.M. (2022). Melanoma Lymph Node Metastases—Moving beyond Quantity in Clinical Trial Design and Contemporary Practice. Front. Oncol..

[13] Sridhara N.G., Sridhara N.G., Li W., Ponnatapura J. (2023). A Rare Radiological Presentation of Pulmonary Metastases from Malignant Melanoma. Radiol. Case Rep..

[14] Obrador E., Salvador R., López-Blanch R., Jihad-Jebbar A., Alcácer J., Benlloch M., Pellicer J.A., Estrela J.M. (2021). Melanoma in the Liver: Oxidative Stress and the Mechanisms of Metastatic Cell Survival. Semin. Cancer Biol..

[15] Provance O.K., Oria V.O., Tran T.T., Caulfield J.I., Zito C.R., Aguirre-Ducler A., Schalper K.A., Kluger H.M., Jilaveanu L.B. (2024). Vascular Mimicry as a Facilitator of Melanoma Brain Metastasis. Cell. Mol. Life Sci..

[16] Gullapalli K., Agarwal P., Mosalem O., Gogineni V., Tikaria R. (2022). Extra-Axial Skeletal Metastasis of Malignant Melanoma: Case Report and Literature Review. Cureus.

[17] Di Raimondo C., Lozzi F., Di Domenico P.P., Campione E., Bianchi L. (2023). The Diagnosis and Management of Cutaneous Metastases from Melanoma. Int. J. Mol. Sci..

[18] Serrao E.M., Costa A.M., Ferreira S., McMorran V., Cargill E., Hough C., Shaw A.S., O’Carrigan B., Parkinson C.A., Corrie P.G. (2022). The Different Faces of Metastatic Melanoma in the Gastrointestinal Tract. Insights Imaging.

[19] Sandru A., Voinea S., Panaitescu E., Blidaru A. (2014). Survival Rates of Patients with Metastatic Malignant Melanoma. J. Med. Life.

[20] van Not O.J., van den Eertwegh A.J.M., Haanen J.B., Blank C.U., Aarts M.J.B., van Breeschoten J., van den Berkmortel F.W.P.J., de Groot J.-W.B., Hospers G.A.P., Ismail R.K. (2024). Improving Survival in Advanced Melanoma Patients: A Trend Analysis from 2013 to 2021. eClinicalMedicine.

[21] Rabbie R., Ferguson P., Molina-Aguilar C., Adams D.J., Robles-Espinoza C.D. (2019). Melanoma Subtypes: Genomic Profiles, Prognostic Molecular Markers and Therapeutic Possibilities. J. Pathol..

[22] Yang T.-T., Yu S., Ke C.-L.K., Cheng S.-T. (2023). The Genomic Landscape of Melanoma and Its Therapeutic Implications. Genes.

[23] Bai X., Kong Y., Chi Z., Sheng X., Cui C., Wang X., Mao L., Tang B., Li S., Lian B. (2017). MAPK Pathway and TERT Promoter Gene Mutation Pattern and Its Prognostic Value in Melanoma Patients: A Retrospective Study of 2793 Cases. Clin. Cancer Res..

[24] Kong Y., Si L., Zhu Y., Xu X., Corless C.L., Flaherty K.T., Li L., Li H., Sheng X., Cui C. (2011). Large-Scale Analysis of KIT Aberrations in Chinese Patients with Melanoma. Clin. Cancer Res..

[25] Carvajal R.D., Antonescu C.R., Wolchok J.D., Chapman P.B., Roman R.-A., Teitcher J., Panageas K.S., Busam K.J., Chmielowski B., Lutzky J. (2011). KIT as a Therapeutic Target in Metastatic Melanoma. JAMA.

[26] Stoff R., Markovic S., McWilliams R.R., Yan Y., Tan W., Seetharam M., Block M.S. (2024). Efficacy and Toxicity of KIT Targeted Therapy in Elderly Patients with Melanoma. J. Clin. Oncol..

[27] Castellani G., Buccarelli M., Arasi M.B., Rossi S., Pisanu M.E., Bellenghi M., Lintas C., Tabolacci C. (2023). BRAF Mutations in Melanoma: Biological Aspects, Therapeutic Implications, and Circulating Biomarkers. Cancers.

[28] Menzies A.M., Haydu L.E., Visintin L., Carlino M.S., Howle J.R., Thompson J.F., Kefford R.F., Scolyer R.A., Long G.V. (2012). Distinguishing Clinicopathologic Features of Patients with V600E and V600K BRAF-Mutant Metastatic Melanoma. Clin. Cancer Res..

[29] Heppt M.V., Siepmann T., Engel J., Schubert-Fritschle G., Eckel R., Mirlach L., Kirchner T., Jung A., Gesierich A., Ruzicka T. (2017). Prognostic Significance of BRAF and NRAS Mutations in Melanoma: A German Study from Routine Care. BMC Cancer.

[30] Vasudevan H., Delley C., Aabedi A., Shukla P., Nguyen M., Morshed R.A., Young J.S., Demaree B., Diwanji D., Hervey-Jumper S.L. (2022). BIOM-02. Mutational analysis and single cell sequencing of melanoma brain metastases reveals braf status correlates with clinical outcome and differential immune populations. Neuro-Oncology.

[31] Zablocka T., Kreismane M., Pjanova D., Isajevs S. (2022). Effects of BRAF V600E and NRAS Mutational Status on the Progression-free Survival and Clinicopathological Characteristics of Patients with Melanoma. Oncol. Lett..

[32] Si L., Zou Z., Zhang W., Fang M., Zhang X., Luo Z., Chen J., Huang G., Zhang P., Cheng Y. (2023). Efficacy and Safety of Tunlametinib in Patients with Advanced NRAS-Mutant Melanoma: A Multicenter, Open-Label, Single-Arm, Phase 2 Study. J. Clin. Oncol..

[33] Zhou L., Wang X., Chi Z., Sheng X., Kong Y., Mao L., Lian B., Tang B., Yan X., Bai X. (2021). Association of NRAS Mutation With Clinical Outcomes of Anti-PD-1 Monotherapy in Advanced Melanoma: A Pooled Analysis of Four Asian Clinical Trials. Front. Immunol..

[34] Guida M., Bartolomeo N., Quaglino P., Madonna G., Pigozzo J., Di Giacomo A.M., Minisini A.M., Tucci M., Spagnolo F., Occelli M. (2021). No Impact of NRAS Mutation on Features of Primary and Metastatic Melanoma or on Outcomes of Checkpoint Inhibitor Immunotherapy: An Italian Melanoma Intergroup (IMI) Study. Cancers.

[35] Cirenajwis H., Lauss M., Ekedahl H., Törngren T., Kvist A., Saal L.H., Olsson H., Staaf J., Carneiro A., Ingvar C. (2017). 1-Mutated Melanoma Tumors Harbor Distinct Clinical and Biological Characteristics. Mol. Oncol..

[36] Nissan M.H., Pratilas C.A., Jones A.M., Ramirez R., Won H., Liu C., Tiwari S., Kong L., Hanrahan A.J., Yao Z. (2014). Loss of NF1 in Cutaneous Melanoma Is Associated with RAS Activation and MEK Dependence. Cancer Res..

[37] Meyer S.N., Simmons E., Studer A.C., Rauen K.A., Kiuru M. (2023). Melanocytic Neoplasms in Neurofibromatosis Type 1: A Systematic Review. Melanoma Res..

[38] Kodali N., Bhattaru A., Blanchard I., Sharma Y., Lipner S.R. (2024). Assessing Melanoma Prognosis: The Interplay between Patient Profiles, Survival, and BRAF, NRAS, KIT, and TWT Mutations in a Retrospective Multi-Study Analysis. Melanoma Res..

[39] Carrera C., Gual A., Díaz A., Puig-Butillé J.A., Noguès S., Vilalta A., Conill C., Rull R., Vilana R., Arguis P. (2017). Prognostic Role of the Histological Subtype of Melanoma on the Hands and Feet in Caucasians. Melanoma Res..

[40] Davis L.E., Shalin S.C., Tackett A.J. (2019). Current State of Melanoma Diagnosis and Treatment. Cancer Biol. Ther..

[41] Santamaria-Barria J.A., Mammen J.M.V. (2022). Surgical Management of Melanoma: Advances and Updates. Curr. Oncol. Rep..

[42] Pham J.P., Joshua A.M., da Silva I.P., Dummer R., Goldinger S.M. (2023). Chemotherapy in Cutaneous Melanoma: Is There Still a Role?. Curr. Oncol. Rep..

[43] Knight A., Karapetyan L., Kirkwood J.M. (2023). Immunotherapy in Melanoma: Recent Advances and Future Directions. Cancers.

[44] Ribas A., Flaherty K.T. (2011). BRAF Targeted Therapy Changes the Treatment Paradigm in Melanoma. Nat. Rev. Clin. Oncol..

[45] Ismail R.K., Suijkerbuijk K.P.M., de Boer A., van Dartel M., Hilarius D.L., Pasmooij A.M.G., van Zeijl M.C.T., Aarts M.J.B., van den Berkmortel F.W.P.J., Blank C.U. (2022). Long-Term Survival of Patients with Advanced Melanoma Treated with BRAF-MEK Inhibitors. Melanoma Res..

[46] Delyon J., Lebbe C., Dumaz N. (2020). Targeted Therapies in Melanoma beyond BRAF: Targeting NRAS-Mutated and KIT-Mutated Melanoma. Curr. Opin. Oncol..

[47] Patel M., Eckburg A., Gantiwala S., Hart Z., Dein J., Lam K., Puri N. (2021). Resistance to Molecularly Targeted Therapies in Melanoma. Cancers.

[48] Krayem M., Ghanem G.E., Van Gestel D. (2022). Recent Advances in Radiosensitivity Determinants in Melanoma. Curr. Opin. Oncol..

[49] Shi W., Ward W.H., Farma J.M. (2017). Radiation Therapy for Melanoma. Cutaneous Melanoma: Etiology and Therapy.

[50] Spatola C., Liardo R.L.E., Milazzotto R., Raffaele L., Salamone V., Basile A., Foti P.V., Palmucci S., Cirrone G.A.P., Cuttone G. (2020). Radiotherapy of Conjunctival Melanoma: Role and Challenges of Brachytherapy, Photon-Beam and Protontherapy. Appl. Sci..

[51] Umebayashi Y., Uyeno K., Tsujii H., Otsuka F. (1995). Proton Radiotherapy for Malignant Melanoma of the Skin. Dermatology.

[52] Dong Y.C., Nieves L.M., Hsu J.C., Kumar A., Bouché M., Krishnan U., Mossburg K.J., Saxena D., Uman S., Kambayashi T. (2023). Novel Combination Treatment for Melanoma: FLASH Radiotherapy and Immunotherapy Delivered by a Radiopaque and Radiation Responsive Hydrogel. Chem. Mater..

[53] Trappetti V., Fazzari J.M., Fernandez-Palomo C., Scheidegger M., Volarevic V., Martin O.A., Djonov V.G. (2021). Microbeam Radiotherapy—A Novel Therapeutic Approach to Overcome Radioresistance and Enhance Anti-Tumour Response in Melanoma. Int. J. Mol. Sci..

[54] Ishiguro A., Ogata D., Okuma K., Kashihara T., Murakami N., Hiki K., Yamakawa K., Jinnai S., Takahashi A., Namikawa K. (2023). Malignant Melanoma Treatment Using Brachytherapy: Two Case Reports and 15 Case Series. J. Dermatol..

[55] Takahashi J., Nagasawa S. (2020). Immunostimulatory Effects of Radiotherapy for Local and Systemic Control of Melanoma: A Review. Int. J. Mol. Sci..

[56] Shi Y., Zhao W., Ding Y., Ge X., Ju M. (2024). Research on the Influence of Radiotherapy-Related Genes on Immune Infiltration, Immunotherapy Response and Prognosis in Melanoma Based on Multi-Omics. Front. Immunol..

[57] Hendrickx A., Cozzio A., Plasswilm L., Panje C.M. (2020). Radiotherapy for Lentigo Maligna and Lentigo Maligna Melanoma—A Systematic Review. Radiat. Oncol..

[58] Wallington D.G., Rashid A.S., Buchwald Z.S., Sudmeier L.J., Khan M.K. (2020). Complete and Durable Response After Radiation Therapy to Primary Tumor Site of a Patient with Metastatic Anorectal Mucosal Melanoma with Oligoprogression on Nivolumab. Adv. Radiat. Oncol..

[59] Chan A.W., Lin H., Yacoub I., Chhabra A.M., Choi J.I., Simone C.B. (2024). Proton Therapy in Uveal Melanoma. Cancers.

[60] Stålhammar G. (2020). Forty-Year Prognosis after Plaque Brachytherapy of Uveal Melanoma. Sci. Rep..

[61] Stålhammar G., Seregard S., Damato B.E., Damato B.E., Singh A.D. (2019). Uveal Melanoma: Brachytherapy. Clinical Ophthalmic Oncology: Uveal Tumors.

[62] Semeniuk O., Yu E., Rivard M.J. (2024). Current and Emerging Radiotherapy Options for Uveal Melanoma. Cancers.

[63] Henderson M.A., Burmeister B.H., Ainslie J., Fisher R., Di Iulio J., Smithers B.M., Hong A., Shannon K., Scolyer R.A., Carruthers S. (2015). Adjuvant Lymph-Node Field Radiotherapy versus Observation Only in Patients with Melanoma at High Risk of Further Lymph-Node Field Relapse after Lymphadenectomy (ANZMTG 01.02/TROG 02.01): 6-Year Follow-up of a Phase 3, Randomised Controlled Trial. Lancet Oncol..

[64] King A.L.O., Lee V., Yu B., Mirza F.N., Zogg C.K., Yang D.X., Tran T., Leventhal J., An Y. (2023). Factors Associated with the Use of Adjuvant Radiation Therapy in Stage III Melanoma. Front. Oncol..

[65] Vuong W., Lin J., Wei R.L. (2017). Palliative Radiotherapy for Skin Malignancies. Ann. Palliat. Med..

[66] Rogers S.J., Puric E., Eberle B., Datta N.R., Bodis S.B. (2019). Radiotherapy for Melanoma: More than DNA Damage. Dermatol. Res. Pract..

[67] Sia J., Szmyd R., Hau E., Gee H.E. (2020). Molecular Mechanisms of Radiation-Induced Cancer Cell Death: A Primer. Front. Cell Dev. Biol..

[68] Mahadevan A., Patel V.L., Dagoglu N. (2015). Radiation Therapy in the Management of Malignant Melanoma. Oncology.

[69] Barranco S.C., Romsdahl M.M., Humphrey R.M. (1971). The Radiation Response of Human Malignant Melanoma Cells Grown in Vitro. Cancer Res..

[70] Dewey D.L. (1971). The Radiosensitivity of Melanoma Cells in Culture. Br. J. Radiol..

[71] Fertil B., Malaise E.P. (1985). Intrinsic Radiosensitivity of Human Cell Lines Is Correlated with Radioresponsiveness of Human Tumors: Analysis of 101 Published Survival Curves. Int. J. Radiat. Oncol. Biol. Phys..

[72] Doss L.L., Memula N. (1982). The Radioresponsiveness of Melanoma. Int. J. Radiat. Oncol. Biol. Phys..

[73] Bentzen S.M., Overgaard J., Thames H.D., Overgaard M., Vejby Hansen P., von der Maase H., Meder J. (1989). Clinical Radiobiology of Malignant Melanoma. Radiother. Oncol. J. Eur. Soc. Ther. Radiol. Oncol..

[74] Overgaard J. (1986). The Role of Radiotherapy in Recurrent and Metastatic Malignant Melanoma: A Clinical Radiobiological Study. Int. J. Radiat. Oncol. Biol. Phys..

[75] Keatts S.A., Salem A.F., Swanson D.M., Farooqi A.S., Bishop A.J., Amaria R.N., McQuade J.L., Glitza Oliva I.C., Diab A., Weiser R. (2024). Durable Local Control with Hypofractionated Radiation Therapy for Unresectable or Metastatic Melanoma. Clin. Transl. Radiat. Oncol..

[76] Fang D., Nguyen T.K., Leishear K., Finko R., Kulp A.N., Hotz S., Van Belle P.A., Xu X., Elder D.E., Herlyn M. (2005). A Tumorigenic Subpopulation with Stem Cell Properties in Melanomas. Cancer Res..

[77] Zabierowski S.E., Herlyn M. (2008). Melanoma Stem Cells: The Dark Seed of Melanoma. J. Clin. Oncol..

[78] Girouard S.D., Murphy G.F. (2011). Melanoma Stem Cells: Not Rare, but Well Done. Lab. Investig..

[79] Roesch A., Fukunaga-Kalabis M., Schmidt E.C., Zabierowski S.E., Brafford P.A., Vultur A., Basu D., Gimotty P., Vogt T., Herlyn M. (2010). A Temporarily Distinct Subpopulation of Slow-Cycling Melanoma Cells Is Required for Continuous Tumor Growth. Cell.

[80] Quintana E., Shackleton M., Sabel M.S., Fullen D.R., Johnson T.M., Morrison S.J. (2008). Efficient Tumour Formation by Single Human Melanoma Cells. Nature.

[81] Civenni G., Walter A., Kobert N., Mihic-Probst D., Zipser M., Belloni B., Seifert B., Moch H., Dummer R., van den Broek M. (2011). Human CD271-Positive Melanoma Stem Cells Associated with Metastasis Establish Tumor Heterogeneity and Long-Term Growth. Cancer Res..

[82] Boyle S.E., Fedele C.G., Corbin V., Wybacz E., Szeto P., Lewin J., Young R.J., Wong A., Fuller R., Spillane J. (2016). CD271 Expression on Patient Melanoma Cells Is Unstable and Unlinked to Tumorigenicity. Cancer Res..

[83] Parmiani G. (2016). Melanoma Cancer Stem Cells: Markers and Functions. Cancers.

[84] Quintana E., Shackleton M., Foster H.R., Fullen D.R., Sabel M.S., Johnson T.M., Morrison S.J. (2010). Phenotypic Heterogeneity among Tumorigenic Melanoma Cells from Patients That Is Reversible and Not Hierarchically Organized. Cancer Cell.

[85] van Niekerk G., Davids L.M., Hattingh S.M., Engelbrecht A.-M. (2017). Cancer Stem Cells: A Product of Clonal Evolution?. Int. J. Cancer.

[86] Peitzsch C., Tyutyunnykova A., Pantel K., Dubrovska A. (2017). Cancer Stem Cells: The Root of Tumor Recurrence and Metastases. Semin. Cancer Biol..

[87] Brinckerhoff C.E. (2017). Cancer Stem Cells (CSCs) in Melanoma: There’s Smoke, but Is There Fire?. J. Cell. Physiol..

[88] Muz B., de la Puente P., Azab F., Azab A.K. (2015). The Role of Hypoxia in Cancer Progression, Angiogenesis, Metastasis, and Resistance to Therapy. Hypoxia.

[89] Spranger S., Bao R., Gajewski T.F. (2015). Melanoma-Intrinsic β-Catenin Signalling Prevents Anti-Tumour Immunity. Nature.

[90] Kreso A., Dick J.E. (2014). Evolution of the Cancer Stem Cell Model. Cell Stem Cell.

[91] Meacham C.E., Morrison S.J. (2013). Tumour Heterogeneity and Cancer Cell Plasticity. Nature.

[92] Yin Q., Shi X., Lan S., Jin H., Wu D. (2021). Effect of Melanoma Stem Cells on Melanoma Metastasis (Review). Oncol. Lett..

[93] Schatton T., Frank M.H. (2009). Antitumor Immunity and Cancer Stem Cells. Ann. N. Y. Acad. Sci..

[94] Speigl L., Janssen N., Weide B., Sinnberg T., Pawelec G., Shipp C. (2023). Putative Cancer Stem Cell Markers Are Frequently Expressed by Melanoma Cells in Vitro and in Situ but Are Also Present in Benign Differentiated Cells. Front. Biosci. Landmark.

[95] Marzagalli M., Moretti R.M., Messi E., Marelli M.M., Fontana F., Anastasia A., Bani M.R., Beretta G., Limonta P. (2018). Targeting Melanoma Stem Cells with the Vitamin E Derivative δ-Tocotrienol. Sci. Rep..

[96] Miranda-Lorenzo I., Dorado J., Lonardo E., Alcala S., Serrano A.G., Clausell-Tormos J., Cioffi M., Megias D., Zagorac S., Balic A. (2014). Intracellular Autofluorescence: A Biomarker for Epithelial Cancer Stem Cells. Nat. Methods.

[97] Castro-Pérez E., Singh M., Sadangi S., Mela-Sánchez C., Setaluri V. (2023). Connecting the Dots: Melanoma Cell of Origin, Tumor Cell Plasticity, Trans-Differentiation, and Drug Resistance. Pigment. Cell Melanoma Res..

[98] Ahmed F., Haass N.K. (2018). Microenvironment-Driven Dynamic Heterogeneity and Phenotypic Plasticity as a Mechanism of Melanoma Therapy Resistance. Front. Oncol..

[99] Wessely A., Steeb T., Berking C., Heppt M.V. (2021). How Neural Crest Transcription Factors Contribute to Melanoma Heterogeneity, Cellular Plasticity, and Treatment Resistance. Int. J. Mol. Sci..

[100] Redmer T., Welte Y., Behrens D., Fichtner I., Przybilla D., Wruck W., Yaspo M.-L., Lehrach H., Schäfer R., Regenbrecht C.R.A. (2014). The Nerve Growth Factor Receptor CD271 Is Crucial to Maintain Tumorigenicity and Stem-Like Properties of Melanoma Cells. PLoS ONE.

[101] Huang F., Santinon F., Flores González R.E., del Rincón S.V. (2021). Melanoma Plasticity: Promoter of Metastasis and Resistance to Therapy. Front. Oncol..

[102] Du Y., Shao H., Moller M., Prokupets R., Tse Y.T., Liu Z.-J. (2019). Intracellular Notch1 Signaling in Cancer-Associated Fibroblasts Dictates the Plasticity and Stemness of Melanoma Stem/Initiating Cells. Stem Cells.

[103] Lee L.M.J., Seftor E.A., Bonde G., Cornell R.A., Hendrix M.J.C. (2005). The Fate of Human Malignant Melanoma Cells Transplanted into Zebrafish Embryos: Assessment of Migration and Cell Division in the Absence of Tumor Formation. Dev. Dyn..

[104] Seftor E.A., Brown K.M., Chin L., Kirschmann D.A., Wheaton W.W., Protopopov A., Feng B., Balagurunathan Y., Trent J.M., Nickoloff B.J. (2005). Epigenetic Transdifferentiation of Normal Melanocytes by a Metastatic Melanoma Microenvironment. Cancer Res..

[105] Hendrix M.J.C., Seftor E.A., Hess A.R., Seftor R.E.B. (2003). Vasculogenic Mimicry and Tumour-Cell Plasticity: Lessons from Melanoma. Nat. Rev. Cancer.

[106] Maniotis A.J., Chen X., Garcia C., DeChristopher P.J., Wu D., Pe’er J., Folberg R. (2002). Control of Melanoma Morphogenesis, Endothelial Survival, and Perfusion by Extracellular Matrix. Lab. Investig..

[107] Folberg R., Hendrix M.J.C., Maniotis A.J. (2000). Vasculogenic Mimicry and Tumor Angiogenesis. Am. J. Pathol..

[108] Al Hmada Y., Brodell R.T., Kharouf N., Flanagan T.W., Alamodi A.A., Hassan S.-Y., Shalaby H., Hassan S.-L., Haikel Y., Megahed M. (2024). Mechanisms of Melanoma Progression and Treatment Resistance: Role of Cancer Stem-like Cells. Cancers.

[109] Wouters J., Vankelecom H., van den Oord J. (2009). Cancer Stem Cells in Cutaneous Melanoma. Expert Rev. Dermatol..

[110] Fattore L., Mancini R., Ciliberto G. (2020). Cancer Stem Cells and the Slow Cycling Phenotype: How to Cut the Gordian Knot Driving Resistance to Therapy in Melanoma. Cancers.

[111] Fisher M.L., Grun D., Adhikary G., Xu W., Eckert R.L. (2017). Inhibition of YAP Function Overcomes BRAF Inhibitor Resistance in Melanoma Cancer Stem Cells. Oncotarget.

[112] Knowles O., Doldan P., Hillier-Richardson I., Lunt S., Youssef G., Gammon L., Mackenzie I.C., Philpott M.P., Rizvi H., Bergamaschi D. (2025). A CD24+CD271+ Melanoma Cancer Stem Cell Possesses Hybrid Characteristics of Its Single Marker Counterparts and Promotes Invasion and Therapeutic Resistance. BMC Biol..

[113] Erfani E., Roudi R., Rakhshan A., Sabet M.N., Shariftabrizi A., Madjd Z. (2016). Comparative Expression Analysis of Putative Cancer Stem Cell Markers CD44 and ALDH1A1 in Various Skin Cancer Subtypes. Int. J. Biol. Markers.

[114] Madjd Z., Erfani E., Gheytanchi E., Moradi-Lakeh M., Shariftabrizi A., Asadi-Lari M. (2016). Expression of CD133 Cancer Stem Cell Marker in Melanoma: A Systematic Review and Meta-Analysis. Int. J. Biol. Markers.

[115] Wu R.-L., Sedlmeier G., Kyjacova L., Schmaus A., Philipp J., Thiele W., Garvalov B.K., Sleeman J.P. (2018). Hyaluronic Acid-CD44 Interactions Promote BMP4/7-Dependent Id1/3 Expression in Melanoma Cells. Sci. Rep..

[116] Ahrens T., Sleeman J.P., Schempp C.M., Howells N., Hofmann M., Ponta H., Herrlich P., Simon J.C. (2001). Soluble CD44 Inhibits Melanoma Tumor Growth by Blocking Cell Surface CD44 Binding to Hyaluronic Acid. Oncogene.

[117] Schatton T., Frank M.H. (2008). Cancer Stem Cells and Human Malignant Melanoma. Pigment. Cell Melanoma Res..

[118] Simbulan-Rosenthal C.M., Haribabu Y., Vakili S., Kuo L.-W., Clark H., Dougherty R., Alobaidi R., Carney B., Sykora P., Rosenthal D.S. (2022). Employing CRISPR-Cas9 to Generate CD133 Synthetic Lethal Melanoma Stem Cells. Int. J. Mol. Sci..

[119] Jamal S.M.E., Alamodi A., Wahl R.U., Grada Z., Shareef M.A., Hassan S.-Y., Murad F., Hassan S.-L., Santourlidis S., Gomez C.R. (2020). Melanoma Stem Cell Maintenance and Chemo-Resistance Are Mediated by CD133 Signal to PI3K-Dependent Pathways. Oncogene.

[120] Luo Y., Dallaglio K., Chen Y., Robinson W.A., Robinson S.E., McCarter M.D., Wang J., Gonzalez R., Thompson D.C., Norris D.A. (2012). ALDH1A Isozymes Are Markers of Human Melanoma Stem Cells and Potential Therapeutic Targets. Stem Cells.

[121] Dinavahi S.S., Gowda R., Gowda K., Bazewicz C.G., Chirasani V.R., Battu M.B., Berg A., Dokholyan N.V., Amin S., Robertson G.P. (2020). Development of a Novel Multi-Isoform ALDH Inhibitor Effective as an Antimelanoma Agent. Mol. Cancer Ther..

[122] Hu Y., Lu L., Xia Y., Chen X., Chang A.E., Hollingsworth R.E., Hurt E., Owen J., Moyer J.S., Prince M.E.P. (2016). Therapeutic Efficacy of Cancer Stem Cell Vaccines in the Adjuvant Setting. Cancer Res..

[123] Frank N.Y., Margaryan A., Huang Y., Schatton T., Waaga-Gasser A.M., Gasser M., Sayegh M.H., Sadee W., Frank M.H. (2005). ABCB5-Mediated Doxorubicin Transport and Chemoresistance in Human Malignant Melanoma. Cancer Res..

[124] Tangella L.P., Arooj M., Deplazes E., Gray E.S., Mancera R.L. (2021). Identification and Characterisation of Putative Drug Binding Sites in Human ATP-Binding Cassette B5 (ABCB5) Transporter. Comput. Struct. Biotechnol. J..

[125] Schatton T., Murphy G.F., Frank N.Y., Yamaura K., Waaga-Gasser A.M., Gasser M., Zhan Q., Jordan S., Duncan L.M., Weishaupt C. (2008). Identification of Cells Initiating Human Melanomas. Nature.

[126] Wilson B.J., Saab K.R., Ma J., Schatton T., Pütz P., Zhan Q., Murphy G.F., Gasser M., Waaga-Gasser A.M., Frank N.Y. (2014). ABCB5 Maintains Melanoma-Initiating Cells through a Proinflammatory Cytokine Signaling Circuit. Cancer Res..

[127] Wang S., Tang L., Lin J., Shen Z., Yao Y., Wang W., Tao S., Gu C., Ma J., Xie Y. (2017). ABCB5 Promotes Melanoma Metastasis through Enhancing NF-κB P65 Protein Stability. Biochem. Biophys. Res. Commun..

[128] Chartrain M., Riond J., Stennevin A., Vandenberghe I., Gomes B., Lamant L., Meyer N., Gairin J.E., Guilbaud N., Annereau J.P. (2012). Melanoma Chemotherapy Leads to the Selection of ABCB5-Expressing Cells. PLoS ONE.

[129] Tu J., Wang J., Tang B., Zhang Z., Han M., Li M., Yu J., Shen L., Zhang M., Ye J. (2022). Expression and Clinical Significance of TYRP1, ABCB5, and MMP17 in Sinonasal Mucosal Melanoma. Cancer Biomark..

[130] Xiao J., Egger M.E., McMasters K.M., Hao H. (2018). Differential Expression of ABCB5 in BRAF Inhibitor-Resistant Melanoma Cell Lines. BMC Cancer.

[131] Aya-Bonilla C., Gray E.S., Manikandan J., Freeman J.B., Zaenker P., Reid A.L., Khattak M.A., Frank M.H., Millward M., Ziman M. (2019). Immunomagnetic-Enriched Subpopulations of Melanoma Circulating Tumour Cells (CTCs) Exhibit Distinct Transcriptome Profiles. Cancers.

[132] Mu X., Zhou Y., Yu Y., Zhang M. (2024). The Roles of Cancer Stem Cells and Therapeutic Implications in Melanoma. Front. Immunol..

[133] Zhang Z., Richmond A., Yan C. (2022). Immunomodulatory Properties of PI3K/AKT/mTOR and MAPK/MEK/ERK Inhibition Augment Response to Immune Checkpoint Blockade in Melanoma and Triple-Negative Breast Cancer. Int. J. Mol. Sci..

[134] Yue L., Huang Z.-M., Fong S., Leong S., Jakowatz J.G., Charruyer-Reinwald A., Wei M., Ghadially R. (2015). Targeting ALDH1 to Decrease Tumorigenicity, Growth and Metastasis of Human Melanoma. Melanoma Res..

[135] Schlaak M., Schmidt P., Bangard C., Kurschat P., Mauch C., Abken H. (2012). Regression of Metastatic Melanoma in a Patient by Antibody Targeting of Cancer Stem Cells. Oncotarget.

[136] Gammaitoni L., Giraudo L., Leuci V., Todorovic M., Mesiano G., Picciotto F., Pisacane A., Zaccagna A., Volpe M.G., Gallo S. (2013). Effective Activity of Cytokine-Induced Killer Cells against Autologous Metastatic Melanoma Including Cells with Stemness Features. Clin. Cancer Res..

[137] Yesmin F., Bhuiyan R.H., Ohmi Y., Yamamoto S., Kaneko K., Ohkawa Y., Zhang P., Hamamura K., Cheung N.-K.V., Kotani N. (2021). Ganglioside GD2 Enhances the Malignant Phenotypes of Melanoma Cells by Cooperating with Integrins. Int. J. Mol. Sci..

[138] Eissler N., Ruf P., Mysliwietz J., Lindhofer H., Mocikat R. (2012). Trifunctional Bispecific Antibodies Induce Tumor-Specific T Cells and Elicit a Vaccination Effect. Cancer Res..

[139] Yvon E., Vecchio M.D., Savoldo B., Hoyos V., Dutour A., Anichini A., Dotti G., Brenner M.K. (2009). Immunotherapy of Metastatic Melanoma Using Genetically Engineered GD2-Specific T Cells. Clin. Cancer Res..

[140] Shidal C., Al-Rayyan N., Yaddanapudi K., Davis K.R. (2016). Lunasin Is a Novel Therapeutic Agent for Targeting Melanoma Cancer Stem Cells. Oncotarget.

[141] Petrachi T., Romagnani A., Albini A., Longo C., Argenziano G., Grisendi G., Dominici M., Ciarrocchi A., Dallaglio K. (2016). Therapeutic Potential of the Metabolic Modulator Phenformin in Targeting the Stem Cell Compartment in Melanoma. Oncotarget.

[142] Yang F., Wei J., Zhang S., Zhang X. (2017). Shrimp miR-S8 Suppresses the Stemness of Human Melanoma Stem-like Cells by Targeting the Transcription Factor YB-1. Cancer Res..

[143] Köseer A.S., Di Gaetano S., Arndt C., Bachmann M., Dubrovska A. (2023). Immunotargeting of Cancer Stem Cells. Cancers.

[144] Dratkiewicz E., Simiczyjew A., Mazurkiewicz J., Ziętek M., Matkowski R., Nowak D. (2021). Hypoxia and Extracellular Acidification as Drivers of Melanoma Progression and Drug Resistance. Cells.

[145] Soltantoyeh T., Akbari B., Karimi A., Mahmoodi Chalbatani G., Ghahri-Saremi N., Hadjati J., Hamblin M.R., Mirzaei H.R. (2021). Chimeric Antigen Receptor (CAR) T Cell Therapy for Metastatic Melanoma: Challenges and Road Ahead. Cells.

[146] Huang R.-X., Zhou P.-K. (2020). DNA Damage Response Signaling Pathways and Targets for Radiotherapy Sensitization in Cancer. Signal Transduct. Target. Ther..

[147] Penninckx S., Pariset E., Cekanaviciute E., Costes S.V. (2021). Quantification of Radiation-Induced DNA Double Strand Break Repair Foci to Evaluate and Predict Biological Responses to Ionizing Radiation. NAR Cancer.

[148] Ji J., Dragojevic S., Callaghan C.M., Smith E.J., Talele S., Zhang W., Connors M.A., Mladek A.C., Hu Z., Bakken K.K. (2024). Differential Distribution of the DNA-PKcs Inhibitor Peposertib Selectively Radiosensitizes Patient-Derived Melanoma Brain Metastasis Xenografts. Mol. Cancer Ther..

[149] Bürkel F., Jost T., Hecht M., Heinzerling L., Fietkau R., Distel L. (2020). Dual mTOR/DNA-PK Inhibitor CC-115 Induces Cell Death in Melanoma Cells and Has Radiosensitizing Potential. Int. J. Mol. Sci..

[150] Sabbah M., Najem A., Vanderkerkhove C., Kert F., Jourani Y., Journe F., Awada A., Van Gestel D., Ghanem G.E., Krayem M. (2023). The Benefit of Co-Targeting PARP-1 and c-Met on the Efficacy of Radiotherapy in Wild Type BRAF Melanoma. Front. Med..

[151] Fröhlich L.M., Niessner H., Sauer B., Kämereit S., Chatziioannou E., Riel S., Sinnberg T., Schittek B. (2023). PARP Inhibitors Effectively Reduce MAPK Inhibitor Resistant Melanoma Cell Growth and Synergize with MAPK Inhibitors through a Synthetic Lethal Interaction In Vitro and In Vivo. Cancer Res. Commun..

[152] Giunta E.F., Belli V., Napolitano S., De Falco V., Vitiello P.P., Terminiello M., Caputo V., Vitale P., Zanaletti N., Ciardiello D. (2020). 13P Synergistic Activity of PARP Inhibitor and ATR Inhibitor in Melanoma Cell Lines May Depend on BRAF-V600 Mutation Status. Ann. Oncol..

[153] Maresca L., Stecca B., Carrassa L. (2022). Novel Therapeutic Approaches with DNA Damage Response Inhibitors for Melanoma Treatment. Cells.

[154] Munshi A., Kurland J.F., Nishikawa T., Tanaka T., Hobbs M.L., Tucker S.L., Ismail S., Stevens C., Meyn R.E. (2005). Histone Deacetylase Inhibitors Radiosensitize Human Melanoma Cells by Suppressing DNA Repair Activity. Clin. Cancer Res..

[155] Kim R., Kwon M., An M., Kim S.T., Smith S.A., Loembé A.B., Mortimer P.G.S., Armenia J., Lukashchuk N., Shah N. (2022). Phase II Study of Ceralasertib (AZD6738) in Combination with Durvalumab in Patients with Advanced/Metastatic Melanoma Who Have Failed Prior Anti-PD-1 Therapy. Ann. Oncol..

[156] Kauffmann A., Rosselli F., Lazar V., Winnepenninckx V., Mansuet-Lupo A., Dessen P., van den Oord J.J., Spatz A., Sarasin A. (2008). High Expression of DNA Repair Pathways Is Associated with Metastasis in Melanoma Patients. Oncogene.

[157] Loureiro J.B., Raimundo L., Calheiros J., Carvalho C., Barcherini V., Lima N.R., Gomes C., Almeida M.I., Alves M.G., Costa J.L. (2021). Targeting P53 for Melanoma Treatment: Counteracting Tumour Proliferation, Dissemination and Therapeutic Resistance. Cancers.

[158] Chin L., Garraway L.A., Fisher D.E. (2006). Malignant Melanoma: Genetics and Therapeutics in the Genomic Era. Genes Dev..

[159] Hocker T., Tsao H. (2007). Ultraviolet Radiation and Melanoma: A Systematic Review and Analysis of Reported Sequence Variants. Hum. Mutat..

[160] Hodis E., Watson I.R., Kryukov G.V., Arold S.T., Imielinski M., Theurillat J.-P., Nickerson E., Auclair D., Li L., Place C. (2012). A Landscape of Driver Mutations in Melanoma. Cell.

[161] Avery-Kiejda K.A., Zhang X.D., Adams L.J., Scott R.J., Vojtesek B., Lane D.P., Hersey P. (2008). Small Molecular Weight Variants of P53 Are Expressed in Human Melanoma Cells and Are Induced by the DNA-Damaging Agent Cisplatin. Clin. Cancer Res..

[162] Satyamoorthy K., Chehab N.H., Waterman M.J., Lien M.C., El-Deiry W.S., Herlyn M., Halazonetis T.D. (2000). Aberrant Regulation and Function of Wild-Type P53 in Radioresistant Melanoma Cells. Cell Growth Differ..

[163] Strasberg Rieber M., Zangemeister-Wittke U., Rieber M. (2001). P53-Independent Induction of Apoptosis in Human Melanoma Cells by a Bcl-2/Bcl-xL Bispecific Antisense Oligonucleotide. Clin. Cancer Res..

[164] Kao W.H., Riker A.I., Kushwaha D.S., Ng K., Enkemann S.A., Jove R., Buettner R., Zinn P.O., Sánchez N.P., Villa J.L. (2011). Upregulation of Fanconi Anemia DNA Repair Genes in Melanoma Compared to Non-Melanoma Skin Cancer. J. Investig. Dermatol..

[165] Longerich S., Li J., Xiong Y., Sung P., Kupfer G.M. (2014). Stress and DNA Repair Biology of the Fanconi Anemia Pathway. Blood.

[166] Krumm A., Barckhausen C., Kücük P., Tomaszowski K.-H., Loquai C., Fahrer J., Krämer O.H., Kaina B., Roos W.P. (2016). Enhanced Histone Deacetylase Activity in Malignant Melanoma Provokes RAD51 and FANCD2-Triggered Drug Resistance. Cancer Res..

[167] Hatton D.H., Mitchell D.L., Strickland P.T., Johnson R.T. (1995). Enhanced Photoproduct Repair: Its Role in the DNA Damage-Resistance Phenotype of Human Malignant Melanoma Cells. Cancer Res..

[168] Jochemsen A.G. (2014). Reactivation of P53 as Therapeutic Intervention for Malignant Melanoma. Curr. Opin. Oncol..

[169] Arnoff T.E., El-Deiry W.S. (2022). MDM2/MDM4 Amplification and CDKN2A Deletion in Metastatic Melanoma and Glioblastoma Multiforme May Have Implications for Targeted Therapeutics and Immunotherapy. Am. J. Cancer Res..

[170] Sachweh M.C.C., Stafford W.C., Drummond C.J., McCarthy A.R., Higgins M., Campbell J., Brodin B., Arnér E.S.J., Laín S. (2015). Redox Effects and Cytotoxic Profiles of MJ25 and Auranofin towards Malignant Melanoma Cells. Oncotarget.

[171] Fröhlich L.M., Makino E., Sinnberg T., Schittek B. (2022). Enhanced Expression of P21 Promotes Sensitivity of Melanoma Cells towards Targeted Therapies. Exp. Dermatol..

[172] Ingelshed K., Spiegelberg D., Kannan P., Påvénius L., Hacheney J., Jiang L., Eisinger S., Lianoudaki D., Lama D., Castillo F. (2022). The MDM2 Inhibitor Navtemadlin Arrests Mouse Melanoma Growth In Vivo and Potentiates Radiotherapy. Cancer Res. Commun..

[173] Budden T., Davey R.J., Vilain R.E., Ashton K.A., Braye S.G., Beveridge N.J., Bowden N.A. (2016). Repair of UVB-Induced DNA Damage Is Reduced in Melanoma Due to Low XPC and Global Genome Repair. Oncotarget.

[174] Biau J., Devun F., Jdey W., Kotula E., Quanz M., Chautard E., Sayarath M., Sun J.-S., Verrelle P., Dutreix M. (2014). A Preclinical Study Combining the DNA Repair Inhibitor Dbait with Radiotherapy for the Treatment of Melanoma. Neoplasia.

[175] Le Tourneau C., Dreno B., Kirova Y., Grob J.J., Jouary T., Dutriaux C., Thomas L., Lebbé C., Mortier L., Saiag P. (2016). First-in-Human Phase I Study of the DNA-Repair Inhibitor DT01 in Combination with Radiotherapy in Patients with Skin Metastases from Melanoma. Br. J. Cancer.

[176] Viallard C., Chezal J.-M., Mishellany F., Ranchon-Cole I., Pereira B., Herbette A., Besse S., Boudhraa Z., Jacquemot N., Cayre A. (2016). Targeting DNA Repair by coDbait Enhances Melanoma Targeted Radionuclide Therapy. Oncotarget.

[177] Thivat E., Rouanet J., Auzeloux P., Sas N., Jouberton E., Levesque S., Billoux T., Mansard S., Molnar I., Chanchou M. (2022). Phase I Study of [131I] ICF01012, a Targeted Radionuclide Therapy, in Metastatic Melanoma: MELRIV-1 Protocol. BMC Cancer.

[178] Crabtree H.G., Cramer W., Murray J.A. (1997). The Action of Radium on Cancer Cells. II.—Some Factors Determining the Susceptibility of Cancer Cells to Radium. Proc. R. Soc. Lond. Ser. B Contain. Pap. Biol. Character.

[179] Gray L.H., Conger A.D., Ebert M., Hornsey S., Scott O.C. (1953). The Concentration of Oxygen Dissolved in Tissues at the Time of Irradiation as a Factor in Radiotherapy. Br. J. Radiol..

[180] D’Aguanno S., Mallone F., Marenco M., Del Bufalo D., Moramarco A. (2021). Hypoxia-Dependent Drivers of Melanoma Progression. J. Exp. Clin. Cancer Res..

[181] Hammond E.M., Asselin M.-C., Forster D., O’Connor J.P.B., Senra J.M., Williams K.J. (2014). The Meaning, Measurement and Modification of Hypoxia in the Laboratory and the Clinic. Clin. Oncol..

[182] Höckel M., Vaupel P. (2001). Tumor Hypoxia: Definitions and Current Clinical, Biologic, and Molecular Aspects. J. Natl. Cancer Inst..

[183] Jeon S., Jeon M., Choi S., Yoo S., Park S., Lee M., Kim I. (2023). Hypoxia in Skin Cancer: Molecular Basis and Clinical Implications. Int. J. Mol. Sci..

[184] Bedogni B., Powell M.B. (2009). Unique Transforming Properties of Notch1 in Human Melanocytes. Pigment. Cell Melanoma Res..

[185] Lartigau E., Randrianarivelo H., Avril M.F., Margulis A., Spatz A., Eschwège F., Guichard M. (1997). Intratumoral Oxygen Tension in Metastatic Melanoma. Melanoma Res..

[186] Kaanders J.H.A.M., Bussink J., van der Kogel A.J. (2004). Clinical Studies of Hypoxia Modification in Radiotherapy. Semin. Radiat. Oncol..

[187] Gong L., Zhang Y., Liu C., Zhang M., Han S. (2021). Application of Radiosensitizers in Cancer Radiotherapy. Int. J. Nanomed..

[188] Tharmalingham H., Hoskin P. (2019). Clinical Trials Targeting Hypoxia. Br. J. Radiol..

[189] Rofstad E.K., Brustad T. (1978). The Radiosensitizing Effect of Metronidazole and Misonidazole (Ro-07-0582) on a Human Malignant Melanoma Grown in the Athymic Mutant Nude Mouse. Br. J. Radiol..

[190] Guichard M., Malaise E.P. (1982). Radiosensitizing Effects of Misonidazole and SR 2508 on a Human Melanoma Transplanted in Nude Mice: Influence on Repair of Potentially Lethal Damage. Int. J. Radiat. Oncol. Biol. Phys..

[191] el Gamoussi R., Stratford I.J., Guichard M. (1993). Relationship between Intracellular Concentration and Radiosensitizing Effect of Pimonidazole and Etanidazole on Two Human Melanoma Cell Lines. Int. J. Radiat. Biol..

[192] Buscà R., Berra E., Gaggioli C., Khaled M., Bille K., Marchetti B., Thyss R., Fitsialos G., Larribère L., Bertolotto C. (2005). Hypoxia-Inducible Factor 1α Is a New Target of Microphthalmia-Associated Transcription Factor (MITF) in Melanoma Cells. J. Cell Biol..

[193] Cairns R.A., Papandreou I., Sutphin P.D., Denko N.C. (2007). Metabolic Targeting of Hypoxia and HIF1 in Solid Tumors Can Enhance Cytotoxic Chemotherapy. Proc. Natl. Acad. Sci. USA.

[194] Martí-Díaz R., Montenegro M.F., Cabezas-Herrera J., Goding C.R., Rodríguez-López J.N., Sánchez-del-Campo L. (2021). Acriflavine, a Potent Inhibitor of HIF-1α, Disturbs Glucose Metabolism and Suppresses ATF4-Protective Pathways in Melanoma under Non-Hypoxic Conditions. Cancers.

[195] O’Connell M.P., Marchbank K., Webster M.R., Valiga A.A., Kaur A., Vultur A., Li L., Herlyn M., Villanueva J., Liu Q. (2013). Hypoxia Induces Phenotypic Plasticity and Therapy Resistance in Melanoma via the Tyrosine Kinase Receptors ROR1 and ROR2. Cancer Discov..

[196] Giuntini G., Monaci S., Cau Y., Mori M., Naldini A., Carraro F. (2020). Inhibition of Melanoma Cell Migration and Invasion Targeting the Hypoxic Tumor Associated CAXII. Cancers.

[197] Li Y., Zhao L., Li X.-F. (2021). Targeting Hypoxia: Hypoxia-Activated Prodrugs in Cancer Therapy. Front. Oncol..

[198] Zhang M., Stevens G. (1998). Effect of Radiation and Tirapazamine (SR-4233) on Three Melanoma Cell Lines. Melanoma Res..

[199] Liu S., Tetzlaff M.T., Wang T., Chen X., Yang R., Kumar S.M., Vultur A., Li P., Martin J.S., Herlyn M. (2017). Hypoxia-Activated Prodrug Enhances Therapeutic Effect of Sunitinib in Melanoma. Oncotarget.

[200] Hegde A., Jayaprakash P., Couillault C.A., Piha-Paul S., Karp D., Rodon J., Pant S., Fu S., Dumbrava E.E., Yap T.A. (2021). A Phase I Dose-Escalation Study to Evaluate the Safety and Tolerability of Evofosfamide in Combination with Ipilimumab in Advanced Solid Malignancies. Clin. Cancer Res..

[201] Kumar P.R., Moore J.A., Bowles K.M., Rushworth S.A., Moncrieff M.D. (2021). Mitochondrial Oxidative Phosphorylation in Cutaneous Melanoma. Br. J. Cancer.

[202] Huang C., Radi R.H., Arbiser J.L. (2021). Mitochondrial Metabolism in Melanoma. Cells.

[203] Neagu M. (2020). Metabolic Traits in Cutaneous Melanoma. Front. Oncol..

[204] Wu L., Hu Z., Huang Y., Yu Y., Liang W., Zheng Q., Huang X., Huang Y., Lu X., Zhao Y. (2016). Radiation Changes the Metabolic Profiling of Melanoma Cell Line B16. PLoS ONE.

[205] Schiliro C., Firestein B.L. (2021). Mechanisms of Metabolic Reprogramming in Cancer Cells Supporting Enhanced Growth and Proliferation. Cells.

[206] Narayanankutty A., Job J.T., Narayanankutty V. (2019). Glutathione, an Antioxidant Tripeptide: Dual Roles in Carcinogenesis and Chemoprevention. Curr. Protein Pept. Sci..

[207] Lin X., Zheng W., Liu J., Zhang Y., Qin H., Wu H., Xue B., Lu Y., Shen P. (2013). Oxidative Stress in Malignant Melanoma Enhances Tumor Necrosis Factor-α Secretion of Tumor-Associated Macrophages That Promote Cancer Cell Invasion. Antioxid. Redox Signal..

[208] Cen D., Gonzalez R.I., Buckmeier J.A., Kahlon R.S., Tohidian N.B., Meyskens F.L. (2002). Disulfiram Induces Apoptosis in Human Melanoma Cells: A Redox-Related Process. Mol. Cancer Ther..

[209] Gellert J., Agardy D.A., Kumar S., Kourtesakis A., Boschert T., Jähne K., Breckwoldt M.O., Bunse L., Wick W., Davies M.A. (2024). Tumoral Interferon Beta Induces an Immune-Stimulatory Phenotype in Tumor-Associated Macrophages in Melanoma Brain Metastases. Cancer Res. Commun..

[210] Li M., Liu D., Lee D., Cheng Y., Baumhover N.J., Marks B.M., Sagastume E.A., Ballas Z.K., Johnson F.L., Morris Z.S. (2021). Targeted Alpha-Particle Radiotherapy and Immune Checkpoint Inhibitors Induces Cooperative Inhibition on Tumor Growth of Malignant Melanoma. Cancers.

[211] Spiotto M., Fu Y.-X., Weichselbaum R.R. (2016). The Intersection of Radiotherapy and Immunotherapy: Mechanisms and Clinical Implications. Sci. Immunol..

[212] Wu B., Zhang B., Li B., Wu H., Jiang M. (2024). Cold and Hot Tumors: From Molecular Mechanisms to Targeted Therapy. Signal Transduct. Target. Ther..

[213] Maleki Vareki S. (2018). High and Low Mutational Burden Tumors versus Immunologically Hot and Cold Tumors and Response to Immune Checkpoint Inhibitors. J. Immunother. Cancer.

[214] Larkin J., Chiarion-Sileni V., Gonzalez R., Grob J.-J., Rutkowski P., Lao C.D., Cowey C.L., Schadendorf D., Wagstaff J., Dummer R. (2019). Five-Year Survival with Combined Nivolumab and Ipilimumab in Advanced Melanoma. N. Engl. J. Med..

[215] Hugo W., Zaretsky J.M., Sun L., Song C., Moreno B.H., Hu-Lieskovan S., Berent-Maoz B., Pang J., Chmielowski B., Cherry G. (2016). Genomic and Transcriptomic Features of Response to Anti-PD-1 Therapy in Metastatic Melanoma. Cell.

[216] Hossain S.M., Gimenez G., Stockwell P.A., Tsai P., Print C.G., Rys J., Cybulska-Stopa B., Ratajska M., Harazin-Lechowska A., Almomani S. (2022). Innate Immune Checkpoint Inhibitor Resistance Is Associated with Melanoma Sub-Types Exhibiting Invasive and de-Differentiated Gene Expression Signatures. Front. Immunol..

[217] Indini A., Massi D., Pirro M., Roila F., Grossi F., Sahebkar A., Glodde N., Bald T., Mandalà M. (2022). Targeting Inflamed and Non-Inflamed Melanomas: Biological Background and Clinical Challenges. Semin. Cancer Biol..

[218] Zaretsky J.M., Garcia-Diaz A., Shin D.S., Escuin-Ordinas H., Hugo W., Hu-Lieskovan S., Torrejon D.Y., Abril-Rodriguez G., Sandoval S., Barthly L. (2016). Mutations Associated with Acquired Resistance to PD-1 Blockade in Melanoma. N. Engl. J. Med..

[219] Tian Y., Kong L., Li Y., Liao Z., Cai X., Deng S., Yang X., Zhang B., Wang Y., Zhang Z. (2023). Dipeptidyl Peptidase 4 Inhibition Sensitizes Radiotherapy by Promoting T Cell Infiltration. OncoImmunology.

[220] Yin G., Guo W., Huang Z., Chen X. (2022). Efficacy of Radiotherapy Combined with Immune Checkpoint Inhibitors in Patients with Melanoma: A Systemic Review and Meta-Analysis. Melanoma Res..

[221] Knispel S., Stang A., Zimmer L., Lax H., Gutzmer R., Heinzerling L., Weishaupt C., Pföhler C., Gesierich A., Herbst R. (2020). Impact of a Preceding Radiotherapy on the Outcome of Immune Checkpoint Inhibition in Metastatic Melanoma: A Multicenter Retrospective Cohort Study of the DeCOG. J. Immunother. Cancer.

[222] Spaas M., Sundahl N., Kruse V., Rottey S., De Maeseneer D., Duprez F., Lievens Y., Surmont V., Brochez L., Reynders D. (2023). Checkpoint Inhibitors in Combination with Stereotactic Body Radiotherapy in Patients with Advanced Solid Tumors: The CHEERS Phase 2 Randomized Clinical Trial. JAMA Oncol..

[223] Zhou H., Tu C., Yang P., Li J., Kepp O., Li H., Zhang L., Zhang L., Zhao Y., Zhang T. (2022). Carbon Ion Radiotherapy Triggers Immunogenic Cell Death and Sensitizes Melanoma to Anti-PD-1 Therapy in Mice. Oncoimmunology.

[224] Marks B.M., Block M., Johnson G.B., Hruska C.B., Pandey M.K., Paulsen A., Schultz M.K., McDonald M.A., Sagastume E.A., Johnson F.L. (2023). Abstract CT104: MC1R Imaging and Histology in the Targeted Imaging of Melanoma for Alpha-Particle Radiotherapy (TIMAR1) Trial. Cancer Res..

[225] Yu J., Green M.D., Li S., Sun Y., Journey S.N., Choi J.E., Rizvi S.M., Qin A., Waninger J.J., Lang X. (2021). Liver Metastasis Restrains Immunotherapy Efficacy via Macrophage-Mediated T Cell Elimination. Nat. Med..

[226] Yu Y., Shaverdian N., Barker C.A., Romesser P.B., Khan A.J., Bakhoum S.F., Riaz N., Powell S.N., Gomez D.R., Lee N.Y. (2021). Effects of Liver Metastases and Liver-Metastasis-Directed Radiotherapy on Response to Immunotherapy. Int. J. Radiat. Oncol. Biol. Phys..

[227] Jagodinsky J.C., Jin W.J., Bates A.M., Hernandez R., Grudzinski J.J., Marsh I.R., Chakravarty I., Arthur I.S., Zangl L.M., Brown R.J. (2021). Temporal Analysis of Type 1 Interferon Activation in Tumor Cells Following External Beam Radiotherapy or Targeted Radionuclide Therapy. Theranostics.

[228] Postow M.A., Callahan M.K., Barker C.A., Yamada Y., Yuan J., Kitano S., Mu Z., Rasalan T., Adamow M., Ritter E. (2012). Immunologic Correlates of the Abscopal Effect in a Patient with Melanoma. N. Engl. J. Med..

[229] Trommer M., Adams A., Celik E., Fan J., Funken D., Herter J.M., Linde P., Morgenthaler J., Wegen S., Mauch C. (2022). Oncologic Outcome and Immune Responses of Radiotherapy with Anti-PD-1 Treatment for Brain Metastases Regarding Timing and Benefiting Subgroups. Cancers.

[230] Meredith P., Sarna T. (2006). The Physical and Chemical Properties of Eumelanin. Pigment. Cell Res..

[231] Slominski R.M., Sarna T., Płonka P.M., Raman C., Brożyna A.A., Slominski A.T. (2022). Melanoma, Melanin, and Melanogenesis: The Yin and Yang Relationship. Front. Oncol..

[232] Chu C.-N., Hu K.-C., Wu R.S.-C., Bau D.-T. (2021). Radiation-Irritated Skin and Hyperpigmentation May Impact the Quality of Life of Breast Cancer Patients after Whole Breast Radiotherapy. BMC Cancer.

[233] Herraiz C., Martínez-Vicente I., Maresca V. (2021). The α-Melanocyte-Stimulating Hormone/Melanocortin-1 Receptor Interaction: A Driver of Pleiotropic Effects beyond Pigmentation. Pigment. Cell Melanoma Res..

[234] Kadekaro A.L., Leachman S., Kavanagh R.J., Swope V., Cassidy P., Supp D., Sartor M., Schwemberger S., Babcock G., Wakamatsu K. (2010). Melanocortin 1 Receptor Genotype: An Important Determinant of the Damage Response of Melanocytes to Ultraviolet Radiation. FASEB J..

[235] Kinnaert E., Morandini R., Simon S., Hill H.Z., Ghanem G., Houtte P.V. (2000). The Degree of Pigmentation Modulates the Radiosensitivity of Human Melanoma Cells. Radiat. Res..

[236] Pak B.J., Lee J., Thai B.L., Fuchs S.Y., Shaked Y., Ronai Z., Kerbel R.S., Ben-David Y. (2004). Radiation Resistance of Human Melanoma Analysed by Retroviral Insertional Mutagenesis Reveals a Possible Role for Dopachrome Tautomerase. Oncogene.

[237] Kinnaert E., Duez P., Morandini R., Dubois J., Van Houtte P., Ghanem G. (2004). Cysteine but Not Glutathione Modulates the Radiosensitivity of Human Melanoma Cells by Affecting Both Survival and DNA Damage. Pigment. Cell Res..

[238] Lee E.-A., Park B.Y., Kang N., Park C.H., Lee W.S. (2022). Abstract 5970: Tumor Vessel Normalization by a Novel Anti-TIE2 Antibody PMC-403 Enhances the Effectiveness of Radiation Therapy on Cancer Therapy. Cancer Res..

[239] Barker C.A., Postow M.A., Khan S.A., Beal K., Parhar P.K., Yamada Y., Lee N.Y., Wolchok J.D. (2013). Concurrent Radiotherapy and Ipilimumab Immunotherapy for Patients with Melanoma. Cancer Immunol. Res..

[240] Mireștean C.C., Iancu R.I., Iancu D.T. (2023). Radiotherapy and Immunotherapy—A Future Partnership towards a New Standard. Appl. Sci..

[241] Capaccione K.M., Doubrovin M., Braumuller B., Leibowitz D., Bhatt N., Momen-Heravi F., Molotkov A., Kissner M., Goldner K., Soffing M. (2022). Evaluating the Combined Anticancer Response of Checkpoint Inhibitor Immunotherapy and FAP-Targeted Molecular Radiotherapy in Murine Models of Melanoma and Lung Cancer. Cancers.

[242] Postow M.A., Knox S.J., Goldman D.A., Elhanati Y., Mavinkurve V., Wong P., Halpenny D., Reddy S.K., Vado K., McCabe D. (2020). A Prospective, Phase 1 Trial of Nivolumab, Ipilimumab, and Radiotherapy in Patients with Advanced Melanoma. Clin. Cancer Res..

[243] Blank C.U., Lucas M.W., Scolyer R.A., van de Wiel B.A., Menzies A.M., Lopez-Yurda M., Hoeijmakers L.L., Saw R.P.M., Lijnsvelt J.M., Maher N.G. (2024). Neoadjuvant Nivolumab and Ipilimumab in Resectable Stage III Melanoma. N. Engl. J. Med..

[244] Mandalà M., Lorigan P., Sergi M.C., Benannoune N., Serra P., Vitale M.G., Giannarelli D., Arance A.M., Couselo E.M., Neyns B. (2024). Combined Immunotherapy in Melanoma Patients with Brain Metastases: A Multicenter International Study. Eur. J. Cancer.

[245] Davidson T.M., Foster N., Lucien F., Markovic S., Dong H., Winters J.L., Park S.S., Orme J.J. (2022). Rescuing Cancer Immunity by Plasma Exchange in Metastatic Melanoma (ReCIPE-M1): Protocol for a Single-Institution, Open-Label Safety Trial of Plasma Exchange to Clear sPD-L1 for Immunotherapy. BMJ Open.

[246] Orme J.J., Zhang H., Lingamaneni P., Kim Y., Lavoie R., Dorr M., Dizona P., Hirdler J., Bering E.A., Gicobi J.K. (2025). Plasma Exchange and Radiation Resensitize Immunotherapy-Refractory Melanoma: A Phase I Trial. Nat. Commun..

[247] D’Andrea M.A., Reddy G.K. (2020). Systemic Antitumor Effects and Abscopal Responses in Melanoma Patients Receiving Radiation Therapy. Oncology.

[248] Pangal D.J., Yarovinsky B., Cardinal T., Cote D.J., Ruzevick J., Attenello F.J., Chang E.L., Ye J., Neman J., Chow F. (2022). The Abscopal Effect: Systematic Review in Patients with Brain and Spine Metastases. Neuro-Oncol. Adv..

[249] Bonnen M.D., Ballo M.T., Myers J.N., Garden A.S., Diaz E.M., Gershenwald J.E., Morrison W.H., Lee J.E., Oswald M.J., Ross M.I. (2004). Elective Radiotherapy Provides Regional Control for Patients with Cutaneous Melanoma of the Head and Neck. Cancer.

[250] Fogarty G.B. (2024). Adjuvant Radiation Therapy in Macroscopic Regional Nodal Melanoma. Cancers.

[251] Melzig C., Golestaneh A.F., Mier W., Schwager C., Das S., Schlegel J., Lasitschka F., Kauczor H.-U., Debus J., Haberkorn U. (2018). Combined External Beam Radiotherapy with Carbon Ions and Tumor Targeting Endoradiotherapy. Oncotarget.

[252] Greene D.P., Shield D.R., Shields C.L., Shields J.A., Servat J.J., Lin C.J., Douglass A.M., Fulco E.A., Levin F. (2014). Cutaneous Melanoma Metastatic to the Orbit: Review of 15 Cases. Ophthal. Plast. Reconstr. Surg..

[253] Kasakovski D., Skrygan M., Gambichler T., Susok L. (2021). Advances in Targeting Cutaneous Melanoma. Cancers.

[254] Liew D.N., Kano H., Kondziolka D., Mathieu D., Niranjan A., Flickinger J.C., Kirkwood J.M., Tarhini A., Moschos S., Lunsford L.D. (2011). Outcome Predictors of Gamma Knife Surgery for Melanoma Brain Metastases. J. Neurosurg..

[255] Khan M., Lin J., Liao G., Tian Y., Liang Y., Li R., Liu M., Yuan Y. (2018). SRS in Combination With Ipilimumab: A Promising New Dimension for Treating Melanoma Brain Metastases. Technol. Cancer Res. Treat..

[256] Chang E.L., Selek U., Hassenbusch S.J.I., Maor M.H., Allen P.K., Mahajan A., Sawaya R., Woo S.Y. (2005). Outcome Variation among “Radioresistant” Brain Metastases Treated with Stereotactic Radiosurgery. Neurosurgery.

[257] Coderre J.A., Kalef-Ezra J.A., Fairchild R.G., Micca P.L., Reinstein L.E., Glass J.D. (1988). Boron Neutron Capture Therapy of a Murine Melanoma. Cancer Res..

[258] Yong Z., Song Z., Zhou Y., Liu T., Zhang Z., Zhao Y., Chen Y., Jin C., Chen X., Lu J. (2016). Boron Neutron Capture Therapy for Malignant Melanoma: First Clinical Case Report in China. Chin. J. Cancer Res..

[259] Sauerwein W.A.G., Sancey L., Hey-Hawkins E., Kellert M., Panza L., Imperio D., Balcerzyk M., Rizzo G., Scalco E., Herrmann K. (2021). Theranostics in Boron Neutron Capture Therapy. Life.

[260] Bryant C.M., Dagan R., Holtzman A.L., Fernandes R., Bunnell A., Mendenhall W.M. (2021). Passively Scattered Proton Therapy for Nonmelanoma Skin Cancer with Clinical Perineural Invasion. Int. J. Part. Ther..

[261] Han J.E., Lozano A., Hasan S., Choi J.I., Chhabra A.M., Tsai H., Mohammed N., Patel S., Katz S., Chang J.H. (2022). Proton Therapy Outcomes for Head and Neck Cutaneous Melanoma: Proton Collaborative Group Analysis. Int. J. Part. Ther..

[262] Saiag P., Molinier R., Roger A., Boru B., Otmezguine Y., Otz J., Valery C.-A., Blom A., Longvert C., Beauchet A. (2022). Efficacy of Large Use of Combined Hypofractionated Radiotherapy in a Cohort of Anti-PD-1 Monotherapy-Treated Melanoma Patients. Cancers.

[263] Okuma T., Furudate S., Kambayashi Y., Hashimoto A., Aiba S., Fujimura T. (2021). Successful Treatment of BRAF/MEK Inhibitor-Resistant Advanced Cutaneous Melanoma with Nivolumab plus Ipilimumab Combination Therapy Followed by Intensity-Modulated Radiotherapy. J. Dermatol..

[264] Krayem M., Sabbah M., Najem A., Wouters A., Lardon F., Simon S., Sales F., Journe F., Awada A., Ghanem G.E. (2019). The Benefit of Reactivating P53 under MAPK Inhibition on the Efficacy of Radiotherapy in Melanoma. Cancers.

[265] Fernandez-Palomo C., Trappetti V., Potez M., Pellicioli P., Krisch M., Laissue J., Djonov V. (2020). Complete Remission of Mouse Melanoma after Temporally Fractionated Microbeam Radiotherapy. Cancers.

[266] Kirova Y.M., Chen J., Rabarijaona L.I., Piedbois Y., Le Bourgeois J.-P. (1999). Radiotherapy as Palliative Treatment for Metastatic Melanoma. Melanoma Res..

[267] Khan M.K., Khan N., Almasan A., Macklis R. (2011). Future of Radiation Therapy for Malignant Melanoma in an Era of Newer, More Effective Biological Agents. OncoTargets Ther..

[268] Lee J., Chang J.S., Roh M.R., Jung M., Lee C.-K., Oh B.H., Chung K.Y., Koom W.S., Shin S.J. (2020). Clinical Outcomes of Immune Checkpoint Blocker Therapy for Malignant Melanoma in Korean Patients: Potential Clinical Implications for a Combination Strategy Involving Radiotherapy. Cancer Res. Treat..

[269] Twyman-Saint Victor C., Rech A.J., Maity A., Rengan R., Pauken K.E., Stelekati E., Benci J.L., Xu B., Dada H., Odorizzi P.M. (2015). Radiation and Dual Checkpoint Blockade Activate Non-Redundant Immune Mechanisms in Cancer. Nature.

[270] Merlino G., Flaherty K., Acquavella N., Day C.-P., Aplin A., Holmen S., Topalian S., Van Dyke T., Herlyn M. (2013). Meeting Report: The Future of Preclinical Mouse Models in Melanoma Treatment Is Now. Pigment. Cell Melanoma Res..

[271] Rebecca V.W., Somasundaram R., Herlyn M. (2020). Pre-Clinical Modeling of Cutaneous Melanoma. Nat. Commun..

[272] Algazi A., Soon C., Daud A. (2010). Cancer Management and Research Dovepress Treatment of Cutaneous Melanoma: Current Approaches and Future Prospects. Cancer Manag. Res..

[273] Fang L. (2024). Research Progress and Future Prospects of Three Oncolytic Viruses in the Treatment of Melanoma. Trans. Mater. Biotechnol. Life Sci..

[274] Shafren D.R., Au G.G., Nguyen T., Newcombe N.G., Haley E.S., Beagley L., Johansson E.S., Hersey P., Barry R.D. (2004). Systemic Therapy of Malignant Human Melanoma Tumors by a Common Cold-Producing Enterovirus, Coxsackievirus A21. Clin. Cancer Res..

[275] Schwertner B., Lindner G., Toledo Stauner C., Klapproth E., Magnus C., Rohrhofer A., Gross S., Schuler-Thurner B., Öttl V., Feichtgruber N. (2021). Nectin-1 Expression Correlates with the Susceptibility of Malignant Melanoma to Oncolytic Herpes Simplex Virus In Vitro and In Vivo. Cancers.

[276] Donnelly O., Harrington K., Melcher A., Pandha H. (2013). Live Viruses to Treat Cancer. J. R. Soc. Med..

[277] Jiang Z., He J., Zhang B., Wang L., Long C., Zhao B., Yang Y., Du L., Luo W., Hu J. (2024). A Potential “Anti-Warburg Effect” in Circulating Tumor Cell-Mediated Metastatic Progression?. Aging Dis..

[278] Bafaloukos D., Gazouli I., Koutserimpas C., Samonis G. (2023). Evolution and Progress of mRNA Vaccines in the Treatment of Melanoma: Future Prospects. Vaccines.

[279] Bidram M., Zhao Y., Shebardina N.G., Baldin A.V., Bazhin A.V., Ganjalikhany M.R., Zamyatnin A.A., Ganjalikhani-hakemi M. (2021). mRNA-Based Cancer Vaccines: A Therapeutic Strategy for the Treatment of Melanoma Patients. Vaccines.

[280] Lim J.Y., Brockstedt D.G., Lord E.M., Gerber S.A. (2014). Radiation Therapy Combined with Listeria Monocytogenes-Based Cancer Vaccine Synergize to Enhance Tumor Control in the B16 Melanoma Model. Oncoimmunology.

[281] Xu J., Mu S., Wang Y., Yu S., Wang Z. (2024). Recent Advances in Immunotherapy and Its Combination Therapies for Advanced Melanoma: A Review. Front. Oncol..

[282] Wilgenhof S., Van Nuffel A.M.T., Corthals J., Heirman C., Tuyaerts S., Benteyn D., De Coninck A., Van Riet I., Verfaillie G., Vandeloo J. (2011). Therapeutic Vaccination with an Autologous mRNA Electroporated Dendritic Cell Vaccine in Patients with Advanced Melanoma. J. Immunother..

[283] Arance Fernandez A.M., Baurain J.-F., Vulsteke C., Rutten A., Soria A., Carrasco J., Neyns B., De Keersmaecker B., Van Assche T., Lindmark B. (2019). A Phase I Study (E011-MEL) of a TriMix-Based mRNA Immunotherapy (ECI-006) in Resected Melanoma Patients: Analysis of Safety and Immunogenicity. J. Clin. Oncol..

[284] Wang Y., Zhang L., Xu Z., Miao L., Huang L. (2018). mRNA Vaccine with Antigen-Specific Checkpoint Blockade Induces an Enhanced Immune Response against Established Melanoma. Mol. Ther..

[285] Khattak A., Weber J.S., Meniawy T., Taylor M.H., Ansstas G., Kim K.B., McKean M., Long G.V., Sullivan R.J., Faries M.B. (2023). Distant Metastasis-Free Survival Results from the Randomized, Phase 2 mRNA-4157-P201/KEYNOTE-942 Trial. J. Clin. Oncol..

[286] Cao Y., Ding S., Hu Y., Zeng L., Zhou J., Lin L., Zhang X., Ma Q., Cai R., Zhang Y. (2024). An Immunocompetent Hafnium Oxide-Based STING Nanoagonist for Cancer Radio-Immunotherapy. ACS Nano.

[287] Ni K., Lan G., Chan C., Quigley B., Lu K., Aung T., Guo N., La Riviere P., Weichselbaum R.R., Lin W. (2018). Nanoscale Metal-Organic Frameworks Enhance Radiotherapy to Potentiate Checkpoint Blockade Immunotherapy. Nat. Commun..

